# Epiperimetric inequalities in the obstacle problem for the fractional Laplacian

**DOI:** 10.1007/s00526-024-02767-9

**Published:** 2024-06-25

**Authors:** Matteo Carducci

**Affiliations:** Scuola Normale Superiore, Piazza dei Cavalieri 7, 56126 Pisa, Italy

**Keywords:** 35R35

## Abstract

Using epiperimetric inequalities approach, we study the obstacle problem $\min \{(-\Delta )^su,u-\varphi \}=0,$ for the fractional Laplacian $(-\Delta )^s$ with obstacle $\varphi \in C^{k,\gamma }(\mathbb {R}^n)$, $k\ge 2$ and $\gamma \in (0,1)$. We prove an epiperimetric inequality for the Weiss’ energy $W_{1+s}$ and a logarithmic epiperimetric inequality for the Weiss’ energy $W_{2m}$. Moreover, we also prove two epiperimetric inequalities for negative energies $W_{1+s}$ and $W_{2m}$. By these epiperimetric inequalities, we deduce a frequency gap and a characterization of the blow-ups for the frequencies $\lambda =1+s$ and $\lambda =2m$. Finally, we give an alternative proof of the regularity of the points on the free boundary with frequency $1+s$ and we describe the structure of the points on the free boundary with frequency 2*m*, with $m\in \mathbb {N}$ and $2\,m\le k.$

## Introduction

### Obstacle problem for the fractional Laplacian

Let $\varphi \in C^{k,\gamma }(\mathbb {R}^n)$, $k\ge 2$ and $\gamma \in (0,1)$, that decays rapidly at infinity, we consider a solution of the obstacle problem for the fractional Laplacian with obstacle $\varphi$, that is a function $u:\mathbb {R}^n\rightarrow \mathbb {R}$ such that

$$\begin{aligned} \min \{(-\Delta )^su, u-\varphi \}=0, \end{aligned}$$

i.e.

1 $$\begin{aligned} {\left\{ \begin{array}{ll} u(x)\ge \varphi (x) &{} \text{ in } \mathbb {R}^n\\ (-\Delta )^s u(x)=0 &{} \text{ in } \{u(x)>\varphi (x)\}\\ (-\Delta )^s u(x)\ge 0 &{} \text{ in } \mathbb {R}^n\\ \end{array}\right. } \end{aligned}$$

with $s\in (0,1)$. The fractional Laplacian $(-\Delta )^s$ defined as

$$\begin{aligned} (-\Delta )^su(x):=c_{n,s}\text{ P.V. }\int _{\mathbb {R}^n}\frac{u(x)-u(y)}{|x-y|^{n+2s}}\,dy, \end{aligned}$$

where $c_{n,s}=2^{2\,s}s\frac{\Gamma (\frac{n+2\,s}{2})}{\Gamma (1-s)}\pi ^{-\frac{n}{2}}$ is a normalization constant.

The aim of the paper is to established the optimal regularity of the solution and to describe the structure and the regularity of the free boundary

$$\begin{aligned} \Gamma (u):=\partial \Lambda (u) \end{aligned}$$

where

$$\begin{aligned} \Lambda (u):=\{x\in \mathbb {R}^n:u(x)=\varphi (x)\} \end{aligned}$$

is the contact set.

### The extension operator $L_a$

To study this problem, we will use the Caffarelli-Silvestre extension of *u*. As in [6], we consider the function ${\widetilde{u}}:\mathbb {R}^n\times \mathbb {R}\rightarrow \mathbb {R}$ satisfying

$$\begin{aligned} {\left\{ \begin{array}{ll} L_a {\widetilde{u}}(X)=0&{} \text{ in } \mathbb {R}^{n+1}_+\\ {\widetilde{u}}(x,y)={\widetilde{u}}(x,-y)&{} \text{ in } \mathbb {R}^{n+1}\\ {\widetilde{u}}(x,0)=u(x) &{} \text{ in } \mathbb {R}^n,\\ \end{array}\right. } \end{aligned}$$

where $$X=(x,y)\in \mathbb {R}^{n+1}_+:=\mathbb {R}^n\times (0,+\infty )$$ and

$$\begin{aligned} L_a{\widetilde{u}}(X)=|y|^{-a}\text{ div}_{x,y}(|y|^a\nabla _{x,y} {\widetilde{u}})=\Delta _x{\widetilde{u}}+\frac{a}{y}\partial _y \widetilde{u}+\Delta _y {\widetilde{u}}. \end{aligned}$$

According to [6] (see also [24]), if we choose $a:=1-2s\in (-1,1)$, we get

$$\begin{aligned} \begin{aligned} (-\Delta )^su(x)=-d_s\lim _{y\rightarrow 0^+}(|y|^a\partial _y \widetilde{u}(x,y)), \end{aligned} \end{aligned}$$

with $d_s>0$, i.e. $(-\Delta )^s$ is a Dirichlet-to-Neumann map for $L_a$. Now, since

$$\begin{aligned} \text{ div}_{x,y}(|y|^a\nabla _{x,y} \widetilde{u})=2\lim _{y\rightarrow 0^+}(|y|^a\partial _y {\widetilde{u}}(x,y))\mathcal {H}^n|_{\{y=0\}} \end{aligned}$$

in distributional sense (see Proposition 9.1), we get that the problem (1) is equivalent to

2 $$\begin{aligned} {\left\{ \begin{array}{ll} {\widetilde{u}}(x,0)\ge \varphi (x) &{} \text{ in } \mathbb {R}^n\\ {\widetilde{u}}(x,y)={\widetilde{u}}(x,-y) &{}\text{ in } \mathbb {R}^{n+1}\\ -L_a {\widetilde{u}}(x,y)=0 &{} \text{ in } \mathbb {R}^{n+1}{\setminus } \{u(x,0)=\varphi (x)\}\\ -L_a {\widetilde{u}}(x,y)\ge 0 &{} \text{ in } \mathbb {R}^{n+1},\\ \end{array}\right. } \end{aligned}$$

where $$\varphi \in C^{k,\gamma }(\mathbb {R}^n)$$, $$k\ge 2$$ and $$\gamma \in (0,1)$$.

In particular, when $s=\frac{1}{2}$, i.e. $a=0$ and $L_a=\Delta$, the problem (2) is the thin obstacle problem (also know as Signorini problem).

Localizing the problem in $B_1\subset \mathbb {R}^{n+1}$, the solution of (2) can be obtained by minimizing the functional

3 $$\begin{aligned} \mathcal {E}(v)=\int _{B_1}|\nabla v|^2|y|^a\,dX \end{aligned}$$

among the admissible functions

$$\begin{aligned} \mathcal {K}_c^\varphi :=\{v\in H^1(B_1,a): v\ge \varphi \text{ on } B'_1,\ v=c \text{ on } \partial B_1,\ v(x,y)=v(x,-y) \}, \end{aligned}$$

where $B_1':=B_1\cap \{y=0\}$ and *c* is the trace on $\partial B_1$ of *u*.

Here we denote by $H^1(\Omega ,a):=H^1(\Omega ,|y|^a)$ the weighted Sobolev space. Similarly, $L^2(\Omega ,a):=L^2(\Omega ,|y|^a)$ is the weighted Lebesgue space.

In the following, with a slight abuse of notation, we denote by *u* the $L_a$-extension in $\mathbb {R}^{n+1}$ of *u*, and we suppose that $u\in H^1_{loc}(\mathbb {R}^{n+1},a)$.

Moreover, with a slight abuse of notation, we also denote by $x_0\in \mathbb {R}^n$ the point $(x_0,0)\in \mathbb {R}^{n}\times \{0\}.$

### Obstacle $\varphi \equiv 0$

We will say that *u* is a solution with 0 obstacle, if solves (2) with $\varphi \equiv 0$, i.e. if *u* satisfies

4 $$\begin{aligned} {\left\{ \begin{array}{ll} {\widetilde{u}}(x,0)\ge 0 &{} \text{ in } \mathbb {R}^n\\ {\widetilde{u}}(x,y)=u(x,-y) &{}\text{ in } \mathbb {R}^{n+1}\\ -L_a {\widetilde{u}}(x,y)=0 &{} \text{ in } \mathbb {R}^{n+1}{\setminus } \{u(x,0)=0\}\\ -L_a {\widetilde{u}}(x,y)\ge 0 &{} \text{ in } \mathbb {R}^{n+1}. \end{array}\right. } \end{aligned}$$

Moreover, we denote by

$$\begin{aligned} \begin{aligned} \mathcal {K}_c:=\{v\in H^1(B_1,a): v\ge 0 \text{ on } B'_1,\ v=c \text{ on } \partial B_1,\ v(x,y)=v(x,-y) \} \end{aligned} \end{aligned}$$

the set of admissible function with $\varphi \equiv 0$, then *u* is a minimum of the functional (3) in the class $\mathcal {K}_c$, with $c=u|_{\partial B_1}$.

### Reduction to 0 obstacle

Let *u* be a solution of (2) with obstacle $\varphi \in C^{k,\gamma }(\mathbb {R}^n)$, $k\ge 2$ and $\gamma \in (0,1)$. Let $q_k^{x_0}(x)$ be the *k*-th Taylor polynomial of $\varphi$ at $x_0\in \Gamma (u)$ and ${\widetilde{q}}_k^{x_0}(x,y)$ be a polynomial of degree *k* and the $L_a$-harmonic extension of $q_k^{x_0}(x)$ (see Lemma 9.3). Then ${\widetilde{q}}_k^{x_0}(x,y)$ solves the following problem

$$\begin{aligned} {\left\{ \begin{array}{ll} L_a {\widetilde{q}}_k^{x_0}(x,y)=0&{} \text{ in } \mathbb {R}^{n+1}\\ {\widetilde{q}}_k^{x_0}(x,y)=\widetilde{q}_k^{x_0}(x,-y)&{} \text{ in } \mathbb {R}^{n+1}\\ {\widetilde{q}}_k^{x_0}(x,0)=q_k^{x_0}(x) &{} \text{ in } \mathbb {R}^n\\ |\varphi (x)-q_k^{x_0}(x)|\le C|x -x_0|^{k+\gamma } &{} \text{ in } \mathbb {R}^n. \end{array}\right. } \end{aligned}$$

We can define

$$\begin{aligned} u^{x_0}(x,y)=u(x,y)-\widetilde{q}_k^{x_0}(x,y)-(\varphi (x)-q_k^{x_0}(x)), \end{aligned}$$

then $u^{x_0}$ solves the following problem

5 $$\begin{aligned} {\left\{ \begin{array}{ll} u^{x_0}(x,0)\ge 0 &{} \text{ in } \mathbb {R}^n\\ u^{x_0}(x,y)=u(x,-y) &{}\text{ in } \mathbb {R}^{n+1}\\ -L_a u^{x_0}(x,y)=h(x,y) &{} \text{ in } \mathbb {R}^{n+1}{\setminus } \{u(x,0)=0\}\\ -L_a u^{x_0}(x,y)\ge h(x,y) &{} \text{ in } \mathbb {R}^{n+1}, \end{array}\right. } \end{aligned}$$

where $$h(x,y)=h^{x_0}(x,y):= \Delta _x(\varphi (x)-q_k^{x_0}(x))$$.

Notice that starting from an obstacle problem with obstacle $\varphi \not \equiv 0$, we have reduced the problem to the case $\varphi \equiv 0$, where the right-hand side in the third and fourth line of (5) is not 0. However, the function $h=h^{x_0}$ is very small near $x_0$. Precisely, by the $C^{k,\gamma }$-regularity of $\varphi$,

6 $$\begin{aligned} |h(x,y)|\le C|x-x_0|^{k+\gamma -2}. \end{aligned}$$

Notice that the function $u^{x_0}$ inherits the local behavior of *u*. In what follows we will study the properties of the functions $u^{x_0}$ at all the points $x_0$ on the boundary $\Gamma (u)$ of the contact set $\Lambda (u)$.

### State of art

In this section we give a brief overview on the state of the art of the obstacle for the fractional Laplacian; for more details we refer to [9] for $s\in (0,1)$, and to [11, 23] for the case $s=\frac{1}{2}$.

The obstacle problem for the fractional Laplacian was studied by Silvestre [25], where it was established the almost-optimal regularity $C^{1,\alpha }$ of the solution for all $\alpha \in (0,s)$, in the case $\varphi \in C^{2,1}(\mathbb {R}^n)$. Moreover, in the same paper, it is proved that if $\varphi \in C^{1,\beta }(\mathbb {R}^n)$, then $u\in C^{1,\alpha }(\mathbb {R}^n)$ for all $\alpha \in (0,\min \{s,\beta \})$.

Later, in [7], Caffarelli, Salsa and Silvestre proved the optimal regularity $C^{1,s}$ of the solution for $\varphi \in C^{2,1}(\mathbb {R}^n)$, thus generalizing the result previously obtained by Athanasopoulos and Caffarelli [1] in the case $s=\frac{1}{2}$ and $\varphi \equiv 0$. They use a modification of the following “pure” Almgren’s frequency function

7 $$\begin{aligned} N^{x_0}(r,u):=\frac{r\int _{ B_r(x_0)} |\nabla u |^2|y|^a\,dX}{\int _{\partial B_r(x_0)} u ^2|y|^a\,d\mathcal {H}^n}, \end{aligned}$$

which is monotone in *r* provided that $\varphi \equiv 0$. Precisely, setting

8 $$\begin{aligned} H^{x_0}(r,v)=\int _{\partial B_r(x_0)} v ^2|y|^a\,d\mathcal {H}^n, \end{aligned}$$

they introduce the following generalized Almgren’s frequency function:

$$\begin{aligned} \begin{aligned} \Phi ^{x_0}(r,v)=(r+Cr^{1+p})\frac{d}{dr}\log \max \{H^{x_0}(r,v),r^{n+a+2(k+\gamma -p)}\} \end{aligned} \end{aligned}$$

with $k=2$, $\gamma =1$ and $p=1$. This function is monotone in *r* also when $\varphi \not \equiv 0$, with $\varphi \in C^{k,\gamma }(\mathbb {R}^n)$. Here we denoted by $v=u^{x_0}$, with $x_0\in \Gamma (u)$, a solution to a similar problem to (5).

As a consequence of the monotonicity of $\Phi ^{x_0}$, they obtain that if $x_0\in \Gamma (u)$ is a free boundary point such that

$$\begin{aligned} \Phi ^{x_0}(0^+,v):=\lim _{r\rightarrow 0^+}\Phi ^{x_0}(r,v)=n+a+2\lambda , \end{aligned}$$

for some $\lambda \in (0,2)$, then the rescalings

$$\begin{aligned} v_{r,x_0}(x,y):=\frac{v(x_0+rx,ry)}{\frac{1}{r^{n+a}}\int _{\partial B_r(x_0)} u ^2|y|^a\,d\mathcal {H}^n}, \end{aligned}$$

converge, up to a subsequence, to a function $v_{0,x_0}$ which is a $\lambda$-homogeneous solution of (4) (with 0 obstacle) (see Lemma 6.1 and Lemma 6.2 in [7]).

Thus, the following set of free boundary points is well-defined:

$$\begin{aligned} \Gamma _\lambda (u):= \{x_0\in \Gamma (u): v_{0,x_0} \text{ is } \lambda \text {-homogeneous}\} \end{aligned}$$

for every $\lambda \in (0,2)$. Moreover, it was shown in [7] that $\Gamma _\lambda (u)=\emptyset$ for all $\lambda \in (0,2){\setminus }\{1+s\}$.

We will call $\Gamma _{1+s}(u)$ the set of regular points and we will denote it by $\text{ Reg }(u)$ since it is known to be locally a $C^{1,\alpha }$ submanifold of dimension $n-1$ (see [7]). This is a generalization of the result of Athanasopoulos, Caffarelli and Salsa obtained in [2] in the case $s=\frac{1}{2}$ and $\varphi \equiv 0$.

In the case $\varphi \equiv 0$, we can consider the “pure” Almgren’s frequency function as in (7), and the free boundary can be decomposed as

$$\begin{aligned} \begin{aligned} \Gamma _\lambda (u)&:=\{x_0\in \Gamma (u): u_{0,x_0} \text{ is } \lambda \text {-homogeneous}\}\\ {}&=\{x_0\in \Gamma (u): N^{x_0}(0^+,u)=\lambda \}\\ {}&=\{x_0\in \Gamma (u): \Phi ^{x_0}(0^+,u)=n+a+2\lambda \}. \end{aligned} \end{aligned}$$

Therefore

$$\begin{aligned} \Gamma (u)=\text{ Reg }(u)\cup \text{ Sing }(u)\cup \text{ Other }(u), \end{aligned}$$

where $\text{ Reg }(u)=\Gamma _{1+s}(u)$ are the regular points, $\text{ Sing }(u)=\bigcup _{m\in \mathbb {N}}\Gamma _{2m}$ are the so-called singular points, and $\text{ Other(u) }$ are all the remaining points in $\Gamma (u)$.

For the singular points, in the case $s=\frac{1}{2}$, in [18], Garofalo and Petrosyan proved that $\text{ Sing }(u)$ is contained in the union of at most countably many submanifolds of class $C^{1}$. In [21], using the monotonicity of the generalized Almgren’s frequency for $k\ge 2$, $\gamma \in (0,1)$ and *p* small enough, Garofalo and Ros-Oton extended the structure of singular set to any $s\in (0,1)$. Indeed in the case $\varphi \in C^{k,\gamma }(\mathbb {R}^n)$, $k\ge 2$ and $\gamma \in (0,1)$, they proved that

$$\begin{aligned} \bigcup _{\{m\in \mathbb {N}:\ 2m\le k\}}\Gamma _{2m}(u) \end{aligned}$$

is contained in the union of countably many submanifolds $C^{1}$, where the bound $2m\le k$ is needed in order to assure the existence of blow-ups. Thus, they improved the result previously obtained in [18] (in the case $s=\frac{1}{2}$), where more regularity of the obstacle $\varphi$ was required.

Moreover, Focardi and Spadaro described the entire free boundary, up to sets of null $\mathcal {H}^{n-1}$ measure, in the case $\varphi \equiv 0$ in [14] and in the case $\varphi \not \equiv 0$, with $\varphi$ suitably smooth and decaying fast at infinity, in [15].

In general case, even $\varphi \in C^\infty$, the free boundary might contain points of infinite contact order, as shown explicitly by Fernández-Real and Ros-Oton in [12]. However, Fernández-Real and co-authors in [12, 16] proved that, “generically”, the set where the free boundary is not smooth is at most $(n-2)$-dimensional and the non-regular set has zero $\mathcal {H}^{n-3-\alpha _0}$ measure for a “generic” solution. This solves the analogue of a conjecture of Schaeffer in $\mathbb {R}^3$ and $\mathbb {R}^4$ for the thin obstacle problem.

An alternative proof of the regularity and structure of the free boundary uses epiperimetric inequalities approach for the Weiss’ energy

9 $$\begin{aligned} W^{x_0}_\lambda (r,u):=\frac{1}{r^{n+a+2\lambda -1}}\int _{B_r(x_0)}|\nabla u|^2|y|^a\,dX-\frac{\lambda }{r^{n+a+2\lambda }}\int _{\partial B_r(x_0)}u^2|y|^a\,d\mathcal {H}^n. \end{aligned}$$

In the case $s=\frac{1}{2}$ and $\varphi \equiv 0$, Garofalo, Petrosyan and Smit Vega Garcia [20] and Focardi and Spadaro [13] proved an epiperimetric inequality for $W_{1+s}$ to deduce the regularity $C^{1,\alpha }$ of the regular points $\text{ Reg }(u)$. In the case $s\in (\frac{1}{2},1)$ and $\varphi \not \equiv 0$, using epiperimetric inequalities approach, the same regularity for $\Gamma _{1+s}(u)$ was established by Garofalo, Petrosyan, Pop and Smit Vega Garcia in [19] (see also [17] for $\varphi \equiv 0$ and $s\in (0,1)$). The regularity of the free boundary $\Gamma _{1+s}(u)$ in the case of more general degenerate elliptic operators for variable coefficients was established recently by Banerjee et al. [3], using again an epiperimetric inequality.

Following epiperimetric inequalities approach, Colombo et al. [8] give an alternative proof of the structure of singular set $\text{ Sing }(u)$ in the case $s=\frac{1}{2}$ and $\varphi \equiv 0$. They improved the regularity of the manifolds that contains the singular set up to $C^{1,\log }$, due to the logarithmic epiperimetric inequality for $W_{2m}$.

### Main results

The goal of this paper is to generalize the epiperimetric inequalities that we already know for the thin obstacle problem $s=\frac{1}{2}$, to any $s\in (0,1)$. With this generalization, we can deduce the previous results of regularity and structure of the free boundary, even for non-zero obstacles $\varphi \not \equiv 0$, with $\varphi \in C^{k,\gamma }(\mathbb {R}^n)$, $k\ge 2$ and $\gamma \in (0,1)$. In particular, we prove an epiperimetric inequality for $W_{1+s}$ and a logarithmic epiperimetric inequality for $W_{2m}$, for each $s\in (0,1)$.

Before we state our main results (Theorems 1.1 and 1.2 below), we recall that

10 $$\begin{aligned} \mathcal {K}_c:=\{v\in H^1(B_1,a): v\ge 0 \text{ on } B'_1,\ v=c \text{ on } \partial B_1,\ v(x,y)=v(x,-y) \} \end{aligned}$$

is the set of admissible function, for each $c\in H^1(\partial B_1,a)$. We will also denote by $W_\lambda (u)$ the Weiss’ energy $W_\lambda ^{x_0}(r,u)$, when $x_0=0$ and $r=1$.

#### Theorem 1.1

(Epiperimetric inequality for $W_{1+s}$) Let $\mathcal {K}_c$ defined in (10) and $z=r^{1+s}c(\theta )\in \mathcal {K}_c$ be the $(1+s)$-homogeneous extension in $\mathbb {R}^{n+1}$ of a function $c\in H^1(\partial B_1,a)$. Therefore there is $\zeta \in \mathcal {K}_c$ such that

$$\begin{aligned} W_{1+s}(\zeta )\le (1-\kappa )W_{1+s}(z), \end{aligned}$$

with $$\kappa =\frac{1+a}{2n+a+5}.$$

#### Theorem 1.2

(Logarithmic epiperimetric inequality for $W_{2m}$) Let $\mathcal {K}_c$ defined in (10) and $z=r^{2m}c(\theta )\in \mathcal {K}_c$ be the 2*m*-homogeneous extension in $\mathbb {R}^{n+1}$ of a function $c\in H^1(\partial B_1,a)$. We also suppose that exists a constant $\Theta >0$ such that

11 $$\begin{aligned} \Vert c\Vert _{L^2(\partial B_1,a)}^2\le \Theta \text{ and } |W_{2m}(z)|\le \Theta , \end{aligned}$$

then there is $\zeta \in \mathcal {K}_c$ such that

$$\begin{aligned} W_{2m}(\zeta )\le W_{2m}(z)(1-\varepsilon \Theta ^{-\beta }|W_{2m}(z)|^\beta ), \end{aligned}$$

with $\beta =\frac{n-1}{n+1}$ and ${\varepsilon }={\varepsilon }(n,m,a)>0$ small enough.

The first inequality was originally proved in [20] and in [13] for $s=\frac{1}{2}$ and generalized to any $s\in (0,1)$ in [19] and in [17]. For the proof, we use an alternative method, that follows the idea in [8], decomposing the datum $c\in H^1(\partial B_1,a)$ in terms of eigenfunctions of $L_a$ restricted to $\partial B_1$.

The second inequality was originally proved in [8] for $s=\frac{1}{2}$, but this is a new result for each $s\in (0,1)$.

Moreover, we will give a proof of two epiperimetric inequalities for negative energies^1^ for $W_{1+s}$ and for $W_{2m}$ (see Theorems 5.1 and 5.2), originally proved for $s=\frac{1}{2}$ in [5] and in [8] respectively. Using this two epiperimetric inequalities for negative energies, together with the first two epiperimetric inequalities in Theorems 1.1 and 1.2, we deduce a frequency gap.

#### Proposition 1.3

(Frequency gap) Let *u* be a solution of (2) and $v=u^{x_0}$ be the solution of (5) with $x_0\in \Gamma (u)$, $\varphi \in C^{k,\gamma }(\mathbb {R}^n)$, $k\ge 2$ and $\gamma \in (0,1)$.

If *v* is $\lambda$-homogeneous with $\lambda <k+\gamma$, then

$$\begin{aligned} \lambda \not \in (2s,1+s)\cup (1+s,2)\cup \bigcup _{m\in \mathbb {N}} \left( (2m-c^-_{m,a})\cup (2m+c^+_{m,a})\right) , \end{aligned}$$

for some constants $c^\pm _{m,a}>0$, that depend only on *n*, *m* and *a*.

In particular, if *u* is a $\lambda$-homogeneous solution of (4) (with 0 obstacle) with $\lambda >0$, then the same conclusion hold for *u*.

Notice that $\lambda \not \in \bigcup _{m\in \mathbb {N}}\left( (2m-c^-_{m,a})\cup (2m+c^+_{m,a})\right)$ is a new result for any $s\in (0,1)$ and it is originally proved in [8] for $s=\frac{1}{2}$ and $\varphi \equiv 0$. Observe that the function $-|y|^{2s}$ is a solution of (4) (with 0 obstacle), then we are able to prove the best frequency gap around $1+s$.

Furthermore, we use the epiperimetric inequalities to deduce a characterization of the $\lambda$-homogeneous solutions of (4) (with 0 obstacle), in the case $\lambda =1+s$ and $\lambda =2m$, as described in the following proposition.

#### Proposition 1.4

Let *u* be a $\lambda$-homogeneous solution of (4) (with 0 obstacle).

1. If $\lambda =1+s$, then $u=Ch_e^s$, for some $C\ge 0$ and $e\in \partial B'_1$, where
  12 $$\begin{aligned} h_e^s(x,y)=\left( s^{-1}(x\cdot e)-\sqrt{(x\cdot e)^2+y^2}\right) \left( \sqrt{(x\cdot e)^2+y^2}+(x\cdot e)\right) ^s.\end{aligned}$$
2. If $\lambda =2m$, then $u=p_{2m}$, for some $p_{2m}$ that is a polynomial 2*m*-homogeneous and $L_a$-harmonic, with $p_{2m}\ge 0$ on $B'_1$.

In particular we characterized the blow-ups of a solution *u* at $x_0\in \Gamma (u)$ with frequency $\lambda =1+s$ or $\lambda =2m.$

Finally we use the epiperimetric inequality for $W_{1+s}$ in Theorem 1.1 and the logarithmic epiperimetric inequality for $W_{2m}$ in Theorem 1.2 to get the regularity and the structure of the free boundary. In particular we prove the regularity of the points on the free boundary with frequency $1+s$, denoted by $\Gamma _{1+s}(u)$, and we describe the structure of the points with frequency 2*m*, denoted by $\Gamma _{2m}(u)$, with $2m\le k.$ See (19) below for the definition of $\Gamma _{\lambda }(u)$.

#### Theorem 1.5

Let *u* be a solution of (2) with obstacle $\varphi \in C^{k,\gamma }(\mathbb {R}^n)$, $k\ge 2$ and $\gamma \in (0,1)$.

1. $\Gamma _{1+s}(u)$ is locally a $C^{1,\alpha }$ submanifold of dimension $n-1$, for some $\alpha >0$, i.e. for all $x_0\in \Gamma _{1+s}(u)$, there is $\rho >0$ and $g:U\subset \mathbb {R}^{n-1}\rightarrow \mathbb {R}$ of class $C^{1,\alpha }$ such that
  $$\begin{aligned} \Gamma _{1+s}(u)\cap B'_\rho (x_0)=\{(x,y)\in \mathbb {R}^{n+1}:x_n=g(x_1,\ldots ,x_{n-1})\}\cap B'_\rho (x_0). \end{aligned}$$
2. $\Gamma _{2m}(u)$ is contained in the union of at most countably many submanifolds of class $C^{1,\log }$, for all $2\,m\le k$. In particular, for such *m*, we have that for every $j\in \{0,\ldots n-1\},$
  $$\begin{aligned} \Gamma _{2m}^j(u):=\{x_0\in \Gamma _{2m}:d_{2m}^{x_0}=j\} \end{aligned}$$
  is locally contained in a $C^{1,\log }$ submanifold of dimension *j*, where $d_{2m}^{x_0}$ is defined in (13) below. In particular, when $\varphi \in C^{\infty }(\mathbb {R}^n)$, the singular sets $\cup _{m\in \mathbb {N}}\Gamma _{2m}^j(u)$ is contained in the union of countably many *j*-dimensional submanifolds of class $C^{1,\log }$. Moreover, the singular set $\text{ Sing }(u)=\cup _{m\in \mathbb {N}}\Gamma _{2\,m}(u)$ is contained in the union of countably many submanifolds of class $C^{1,\log }$.

Here we have defined

13 $$\begin{aligned} d_{2m}^{x_0}:=\text{ dim }\{\xi \in \mathbb {R}^{n}:\xi \cdot \nabla _xp_{2m}^{x_0}(x,0)\equiv 0\} \end{aligned}$$

to be the dimension of the “tangent plane”, where $p_{2m}^{x_0}$ is the unique homogeneous blow-up (see Proposition 1.4) of *u* at $x_0\in \Gamma _{2m}(u)$.

In particular, we improve the regularity of submanifolds that contain $\Gamma _{2m}^j(u)$ from $C^{1}$ (proved in [21]) to $C^{1,\log }$.

### Structure of the paper

The paper is organized as follows.

- In Sect. 2, we recall the generalized Almgren’s frequency function, the Weiss’ energy *W* for 0 obstacle and the Weiss’ energy ${\widetilde{W}}$ for non-zero obstacle. We also introduce the operator $L_a^S$, i.e. $L_a$ restricted to $\partial B_1$, its eigenfunctions and its relation to the Weiss’ energy *W*. Finally, we recall the properties of the function $h_e^s$ defined in (12).
- In Sect. 3, we prove Theorem 1.1, i.e. the epiperimetric inequality for $W_{1+s}$.
- In Sect. 4, we prove Theorem 1.2, i.e. the logarithmic epiperimetric inequality for $W_{2m}$.
- In Sect. 5, we state and prove two epiperimetric inequalities for negative energies $W_{1+s}$ and $W_{2m}$ (Theorems 5.1 and 5.2).
- In Sect. 6, using the four epiperimetric inequalities proved in the previous sections, we establish a frequency gap in Proposition 1.3.
- In Sect. 7, using Theorems 1.1 and 1.2, we prove Proposition 1.4, i.e. the characterization of the $\lambda$-homogeneous solutions of (4) (with 0 obstacle), in the case $\lambda =1+s$ and $\lambda =2m$.
- In Sect. 8, we use Theorems 1.1 and 1.2 to prove our main result on the regularity and the structure of the free boundary (Theorem 1.5).

## Preliminaries

### Generalized Almgren’s frequency function

The original generalized Almgren’s frequency function in [7] must be modified in the case $\varphi \in C^{k,\gamma }(\mathbb {R}^n)$, as in the following proposition.

We follow [21], but a similar generalized Almgren’s frequency can be found in [7] or in [4].

First we recall the function $H^{x_0}(r):=H^{x_0}(r,v)$ as in (8), with $v=u^{x_0}$. We drop the dependence on $x_0$ if it is not ambiguous.

#### Proposition 2.1

(Monotonicity of generalized Almgren’s frequency) Let *u* be a solution of (2) and $v=u^{x_0}$ be the solution of (5) with $x_0\in \Gamma (u)$, $\varphi \in C^{k,\gamma }(\mathbb {R}^n)$, $k\ge 2$ and $\gamma \in (0,1)$. We define

$$\begin{aligned} \begin{aligned} \Phi ^{x_0}(r,v):=(r+Cr^{1+p})\frac{d}{dr}\log \max \{H^{x_0}(r),r^{n+a+2(k+\gamma -p)}\}, \end{aligned} \end{aligned}$$

for $p\in (0,\gamma )$ and *C* large enough. Therefore, there is $r_0>0$ such that the function $r\mapsto \Phi ^{x_0}(r,v)$ is monotone increasing for $r\in (0,r_0)$.

#### Proof

The proof is technical and we skip it, since it is standard and proved in many variations. We refer to Proposition 6.1 in [21] for the complete proof. $\square$

By the previous proposition, if $v=u^{x_0}$, it is well-defined

$$\begin{aligned} \Phi ^{x_0}(0^+,v):=\lim _{r\rightarrow 0^+}\Phi ^{x_0}(r,v), \end{aligned}$$

for $$x_0\in \Gamma (u)$$.

In the case of obstacle $\varphi \equiv 0$, if the “pure” Almgren’s frequency $N^{x_0}(0^+,u)=\lambda$, with $N^{x_0}$ as in (7), then we can consider a blow-up of *u* at $x_0$, that is a $\lambda$-homogeneous solution (see [2]).

We want a similar result for $\varphi \in C^{k,\gamma }(\mathbb {R}^n)$. Precisely, we will show that if $\Phi ^{x_0}(0^+,v)=n+a+2\lambda$, then the blow-up of *v* at $x_0$ is a $\lambda$-homogeneous solution. But this is only true if $\lambda <k+\gamma$, and this is the motivation for defining a new generalized Almgren’s frequency, which was originally introduced in [7].

To prove this result, we need the following two lemmas from [21].

#### Lemma 2.2

Let *u* be a solution of (2) and $v=u^{x_0}$ be the solution of (5) with $x_0\in \Gamma (u)$, $\varphi \in C^{k,\gamma }(\mathbb {R}^n)$, $k\ge 2$ and $\gamma \in (0,1)$. If $\Phi ^{x_0}(0^+,v)=n+a+2\lambda$ with $\lambda <k+\gamma$, then

14 $$\begin{aligned} H^{x_0}(r,v)\le Cr^{n+a+2\lambda } \end{aligned}$$

for all $r\in (0,r_0).$ Moreover, for every ${\varepsilon }>0$ there is $r_{\varepsilon }$ such that

15 $$\begin{aligned} H^{x_0}(r,v)\ge Cr^{n+a+2(\lambda +{\varepsilon })} \end{aligned}$$

for all $r\in (0,r_{\varepsilon })$. In particular, $\Phi ^{x_0}(0^+,v)$ does not depend on $p\in (0,\gamma )$.

#### Proof

The proof of the first part follows by the monotonicity of generalized Almgren’s frequency and an integration from *r* to $r_0$. The second part is similar. See Lemma 6.4 in [21] for more details. $\square$

Now we proceed with the second lemma.

#### Lemma 2.3

Let *u* be a solution of (2) and $v=u^{x_0}$ be the solution of (5) with $x_0\in \Gamma (u)$, $\varphi \in C^{k,\gamma }(\mathbb {R}^n)$, $k\ge 2$ and $\gamma \in (0,1)$. If $\Phi ^{x_0}(0^+,v)=n+a+2\lambda$ with $\lambda <k+\gamma$, then

16 $$\begin{aligned} \left|\int _{B_r(x_0)}vh|y|^a\,d\mathcal {H}^n\right|\le Cr^{n+a+\lambda +k+\gamma -1}. \end{aligned}$$

#### Proof

By (6), we obtain

$$\begin{aligned}\begin{aligned} \left|\int _{B_r(x_0)}vh|y|^a\,d\mathcal {H}^n\right|&\le Cr^{k+\gamma -2}\int _{B_r}|v||y|^a\,dX\\&\le Cr^{k+\gamma -2}\left( \int _{B_r(x_0)}|y|^a\,dX\right) ^\frac{1}{2}\left( \int _{B_r(x_0)}v^2|y|^a\right) ^\frac{1}{2}\\&\le Cr^{k+\gamma -2}r^{\frac{n+a+1}{2}}r^{\frac{n+a+2\lambda +1}{2}} =Cr^{n+a+\lambda +k+\gamma -1}, \end{aligned} \end{aligned}$$

where in the last inequality we used (14). $\square$

Now we are ready to prove the existence of a $\lambda$-homogeneous blow-up, when $\lambda <k+\gamma$. We denote by

17 $$\begin{aligned} C^{1,\alpha }_a(\Omega ):=\{w\in C^1(\Omega ):\Vert w\Vert _{C^{1,\alpha }_a(\Omega )}<+\infty \}, \end{aligned}$$

where

$$\begin{aligned} \begin{aligned} \Vert w\Vert _{C^{1,\alpha }_a(\Omega )}:=\Vert w\Vert _{C^{\alpha }(\Omega )}+\Vert \nabla _x w\Vert _{C^{\alpha }(\Omega )}+\Vert |y|^a\partial _y w\Vert _{C^{\alpha }(\Omega )}. \end{aligned} \end{aligned}$$

#### Proposition 2.4

Let *u* be a solution of (2) and $v=u^{x_0}$ be the solution of (5) with $x_0\in \Gamma (u)$, $\varphi \in C^{k,\gamma }(\mathbb {R}^n)$, $k\ge 2$ and $\gamma \in (0,1)$. If $\Phi ^{x_0}(0^+,v)=n+a+2\lambda$ with $\lambda <k+\gamma$, then, up to subsequences, the rescalings

18 $$\begin{aligned} v_{r,x_0}(x,y):=\frac{v(x_0+rx,ry)}{\frac{1}{r^{n+a}}\int _{\partial B_r(x_0)} v ^2|y|^a\,d\mathcal {H}^n} \end{aligned}$$

converge in $C^{1,\alpha }_a(B_R^+)$ for all $R>0$ to a blow-up $v_{0,x_0}$, as $r\rightarrow 0^+$, which is a solution of (4) (with 0 obstacle) and it is $\lambda$-homogeneous.

#### Proof

We can proceed as in the proof of Proposition 6.6 in [21].

We want to point out that, since the values of $\Phi ^{x_0}(0^+,v)$ do not depend on $p\in (0,\gamma )$, by Lemma 2.2, we can choose $p\in (0,\gamma )$ small enough such that

$$\begin{aligned} \lambda +{\varepsilon }<k+\gamma -p, \end{aligned}$$

for some ${\varepsilon }>0$. Hence there is $r_1>0$ such that $H(r)\ge r^{n+a+2(k+\gamma -p)}$ for all $r\in (0,r_1)$, by (15).

In particular, the monotonicity of the function $r\mapsto r(1+Cr^p)\frac{H'(r)}{H(r)}$ is equivalent to the monotonicity of the function $r\mapsto \Phi ^{x_0}(r,v)$ for *r* small enough. $\square$

Notice that the blow-up $v_{0,x_0}$ is not a priori unique. Still, all the blow-ups at $x_0$ have the same homogeneity. Therefore, for $\lambda <k+\gamma$, the following set is well-defined:

19 $$\begin{aligned} \Gamma _\lambda (u):=\{x_0\in \Gamma (u):v_{0,x_0} \text{ is } \lambda \text {-homogeneous}\}. \end{aligned}$$

Equivalently, $\Gamma _\lambda (u)$ is the set of all points $x_0\in \Gamma (u)$ such that $\Phi ^{x_0}(0^+,v)=n+a+2\lambda$, with $\lambda <k+\gamma$.

#### Remark 2.5

As in the proof of the last proposition, we get

20 $$\begin{aligned} \Phi ^{x_0}(r,v)=(1+Cr^p)\left( n+a+2N^{x_0}(r,v) -2\frac{r}{H^{x_0}(r)}\int _{B_{Rr}(x_0)}vh|y|^a\,dX\right) , \end{aligned}$$

for *r* small enough. Then, by (15) and (16), we obtain

$$\begin{aligned} \Phi ^{x_0}(0^+,v)=n+a+2N^{x_0}(0^+,v). \end{aligned}$$

### Properties of the Weiss’ energy ${\widetilde{W}}$

In the case $\varphi \not \equiv 0$, we can consider the following Weiss’ energy, which is a small modification of the Weiss’ energy *W* for the obstacle $\varphi \equiv 0$, defined in (9). Precisely, we define

$$\begin{aligned}\begin{aligned} {\widetilde{W}}^{x_0}_\lambda (r,v)=W^{x_0}_\lambda (r,v)+\frac{1}{r^{n+a+2\lambda -1}}\int _{B_r(x_0)}vh|y|^a\,dX \end{aligned} \end{aligned}$$

and we drop the dependence on $x_0$ if $x_0=0$.

#### Lemma 2.6

Let *u* be a solution of (2) and $v=u^{x_0}$ be the solution of (5) with $x_0\in \Gamma (u)$, $\varphi \in C^{k,\gamma }(\mathbb {R}^n)$, $k\ge 2$ and $\gamma \in (0,1)$. If

$$\begin{aligned} \mathcal {I}(r):=\int _{\partial B_r(x_0)} v \partial _{\nu }v|y|^a\,d\mathcal {H}^n=\int _{B_r(x_0)} |\nabla v |^2|y|^a\,dX+\int _{B_r(x_0)} vh|y|^a\,dX, \end{aligned}$$

then

21 $$\begin{aligned} H'(r)=\frac{n+a}{r}H(r)+2\mathcal {I}(r), \end{aligned}$$

and

22 $$\begin{aligned} \begin{aligned} \mathcal {I}'(r)&=\frac{n+a-1}{r}\mathcal {I}(r) -\frac{n+a-1}{r}\int _{B_r(x_0)} vh|y|^a\,dX\\&\quad +2\int _{\partial B_r(x_0)} (\partial _{\nu }v)^2|y|^a\, d\mathcal {H}^n-2\int _{ B_r(x_0)}h\partial _{\nu }v|y|^a\,dX\\ {}&\quad +\int _{\partial B_r(x_0)}vh|y|^a\,d\mathcal {H}^n. \end{aligned} \end{aligned}$$

#### Proof

We give only the idea of the proof; for more details we refer to [19]. First we can change the variables in the expression of *H*(*r*) and with a standard computation we get (21).

Now, if $X=(x,y)$, then using

$$\begin{aligned} \text{ div }\left( |y|^a\frac{|\nabla v|^2}{2}X-|y|^a\nabla v(X\cdot \nabla v)\right) =\frac{n+a-1}{2}|y|^a|\nabla v|^2-|y|^ah(X\cdot \nabla v) \end{aligned}$$

and an integration by parts, we get (22). $\square$

We next show that also the Weiss’ energy ${\widetilde{W}}$ satisfies a monotonicity formula.

#### Proposition 2.7

(Monotonicity of the Weiss’ energy ${\widetilde{W}}$) Let *u* be a solution of (2) and $v=u^{x_0}$ be the solution of (5) with $x_0\in \Gamma (u)$, $\varphi \in C^{k,\gamma }(\mathbb {R}^n)$, $k\ge 2$ and $\gamma \in (0,1)$. If $\lambda <k+\gamma$, then there exists contants $C,r_0>0$ such that for all $x_0\in \Gamma (u)$ and for all $r\in (0,r_0)$, it holds

23 $$\begin{aligned} \frac{d}{dr}\left( {\widetilde{W}}^{x_0}_\lambda (r,v)+Cr^{k+\gamma -\lambda }\right) \ge \frac{2}{r^{n+a+2\lambda +1}}\int _{\partial B_r(x_0)} (\nabla v\cdot x -\lambda v)^2|y|^a\,d\mathcal {H}^n, \end{aligned}$$

i.e. the function $r\mapsto {\widetilde{W}}^{x_0}_\lambda (r,v)+Cr^{k+\gamma -\lambda }$ is increasing for all $r\in (0,r_0)$.

#### Proof

The original proof is done in [19], Proposition 3.5, in the case $\lambda =1+s$ and with a different exponent for *r* in left-hand side. We generalize this result to any $\lambda$ and to any $\varphi \in C^{k,\gamma }(\mathbb {R}^n)$. $\square$

Since we have

$$\begin{aligned} \begin{aligned} \frac{d}{dr}{\widetilde{W}}^{x_0}_\lambda (r,v)&=-\frac{n+a+2\lambda -1}{r^{n+a+2\lambda }} \mathcal {I}(r)+\frac{\mathcal {I}'(r)}{r^{n+a+2\lambda -1}} +\lambda \frac{n+a+2\lambda }{r^{n+a+2\lambda +1}}H(r)\\ {}&\quad -\frac{\lambda }{r^{n+a+2\lambda }}H'(r), \end{aligned} \end{aligned}$$

by (21) and (22), we obtain

$$\begin{aligned}\begin{aligned} \frac{d}{dr}{\widetilde{W}}^{x_0}_\lambda (r,v)&=\frac{2}{r^{n+a+2\lambda +1}}\int _{\partial B_r}(\nabla v\cdot x -\lambda v)^2|y|^a\,d\mathcal {H}^n\\ {}&\quad -\frac{n+a-1}{r^{n+a+2\lambda }}\int _{B_r(x_0)} vh|y|^a\,dX-\frac{2}{r^{n+a+2\lambda -1}}\int _{ B_r(x_0)}h\partial _{\nu }v|y|^a\,dX\\ {}&\quad +\frac{1}{r^{n+a+2\lambda -1}}\int _{\partial B_r(x_0)}vh|y|^a\,d\mathcal {H}^n. \end{aligned} \end{aligned}$$

Now, we can use (16) to get

$$\begin{aligned} \begin{aligned}&\left|\frac{n+a-1}{r^{n+a+2\lambda }}\int _{B_r(x_0)} vh|y|^a\,dX\right|+\left|\frac{2}{r^{n+a+2\lambda -1}}\int _{ B_r(x_0)}h\partial _{\nu }v|y|^a\,dX\right|\\ {}&+\left|\frac{1}{r^{n+a+2\lambda -1}}\int _{\partial B_r(x_0)}vh|y|^a\,d\mathcal {H}^n\right|\le C r^{k+\gamma -\lambda -1} =C \frac{d}{dr}r^{k+\gamma -\lambda }, \end{aligned} \end{aligned}$$

which proves the claim.

#### Proposition 2.8

Let *u* be a solution of (2) and $v=u^{x_0}$ be the solution of (5) with $x_0\in \Gamma (u)$, $\varphi \in C^{k,\gamma }(\mathbb {R}^n)$, $k\ge 2$ and $\gamma \in (0,1)$. If $\lambda <k+\gamma$ and $x_0\in \Gamma _{\lambda }(u)$, then

24 $$\begin{aligned} {\widetilde{W}}^{x_0}_\lambda (0^+,v)=0. \end{aligned}$$

In particular, using (23), we get

25 $$\begin{aligned} {\widetilde{W}}^{x_0}_\lambda (r,v)\ge -Cr^{k+\gamma -\lambda } \end{aligned}$$

for all $$r\in (0,r_0)$$.

#### Proof

We can write

$$\begin{aligned} {\widetilde{W}}^{x_0}(r,v)=\frac{H^{x_0}(r)}{r^{n+a+2\lambda }}\left( r\frac{\mathcal {I} ^{x_0}(r)}{H^{x_0}(r)}-\lambda \right) . \end{aligned}$$

Hence, using (15) and Remark 2.5, we get

$$\begin{aligned} \begin{aligned} \lim _{ r\rightarrow 0^+}r\frac{\mathcal {I}^{x_0}(r,v)}{H^{x_0}(r)}&=\lim _{ r\rightarrow 0^+}\left( N^{x_0}(r)-\frac{r}{H^{x_0}(r)} \int _{B_{r}(x_0)}vh|y|^a\,dX\right) \\&=N^{x_0}(0^+,v) =\frac{\Phi ^{x_0}(0^+,v)-(n+a)}{2} =\lambda , \end{aligned} \end{aligned}$$

which concludes the proof. $\square$

### Homogeneous rescalings and homogeneous blow-up

The sequence (18) has good rescaling properties with respect to the Almgren’s frequency function. Now we consider another sequence of rescalings, the homogeneous rescalings, which have good rescaling properties with respect to the Weiss’ energy.

#### Proposition 2.9

Let *u* be a solution of (2) and $v=u^{x_0}$ be the solution of (5) with $x_0\in \Gamma (u)$, $\varphi \in C^{k,\gamma }(\mathbb {R}^n)$, $k\ge 2$ and $\gamma \in (0,1)$. Suppose that $\Phi ^{x_0}(0^+,v)=n+a+2\lambda$ with $\lambda <k+\gamma$. Let $v^{(\lambda )}_{r,x_0}$ be the homogeneous rescalings of *v* at $x_0\in \Gamma (u)$, defined as

26 $$\begin{aligned} v^{(\lambda )}_{r,x_0}(x,y):=\frac{v(x_0+rx,ry)}{r^\lambda }. \end{aligned}$$

Then, up to a subsequence, the homogeneous rescalings converge in $C^{1,\alpha }_a(B_R^+)$ for all $R>0$ (defined in (17)), as $r\rightarrow 0^+$, to a blow-up $v^{(\lambda )}_{0,x_0}$, which is $\lambda$-homogeneous and is a solution to (4) (with 0 obstacle).

#### Proof

By the Poincaré inequality (70), in order to show the boundness of $v_r$ in $H^1(B_R,a)$, it is sufficient to prove the boundness of $v_r$ in $L^2(\partial B_R,a)$ and the boundness of $|\nabla v_r|$ in $L^2(B_R,a)$. The first bound follows by (14). In fact

$$\begin{aligned} \Vert v_r\Vert ^2_{L^2(\partial B_R,a)}=H(R,v_r) =\frac{H^{x_0}(Rr,v)}{r^{n+a+2\lambda }}\le C(R). \end{aligned}$$

Also the second bound follows from (14):

$$\begin{aligned}\begin{aligned} \int _{B_R}|\nabla v_r|^2 |y|^a\,dX&=\frac{1}{r^{n+a+2\lambda -1}}\int _{B_{Rr}(x_0)}|\nabla v |^2|y|^a\,dX\\ {}&\le C(R) \frac{Rr}{H^{x_0}(Rr)}\int _{B_{Rr}(x_0)}|\nabla v|^2 |y|^a\,dX\\ {}&=C(R)\Biggl ( N^{x_0}(Rr)-\frac{Rr}{H^{x_0}(Rr)}\int _{B_{Rr}(x_0)}vh|y|^a\,dX\\&\quad +\frac{Rr}{H^{x_0}(Rr)}\int _{B_{Rr}(x_0)}vh|y|^a\,dX\Biggl )\le C(R), \end{aligned} \end{aligned}$$

where in the last inequality we used (15), (16) and the monotonicity of the function $r\mapsto \Phi ^{x_0}(r,v)$. Thus, for *r* small enough,

$$\begin{aligned} r\frac{\mathcal {I}(r)}{H(r)}\le C, \end{aligned}$$

as in the proof of Proposition 2.4.

Hence, up to subsequences, $v_r$ converges to some $v_0$ weakly in $H^1(B_R,a)$ and

27 $$\begin{aligned} \begin{aligned} L_a(v_r(x,y))&=\frac{r^{2}}{r^\lambda }L_av(x_0+rx,ry) \le \frac{r^{2}}{r^\lambda }r^{k+\gamma -2}|x|^{k+\gamma -2}\\ {}&=r^{k+\gamma -\lambda }|x|^{k+\gamma -2} \end{aligned} \end{aligned}$$

by (16), with an equality in $\mathbb {R}^{n+1}{\setminus }(\{v_r=0\}\cap \{y=0\})$. Therefore, we can use the estimate in [7] (Proposition 4.3 and Lemma 4.4) to get the convergence in $C^{1,\alpha }_a$, since $v_r$ are solutions of (5), where the right-hand sides in the third and the fourth lines are as in (27). Moreover, $v_0$ is a solution of (4) (with 0 obstacle), since we can send $r\rightarrow 0^+$ in (27).

Finally, we show that $v_0$ is homogeneous. Indeed, by (23), we have that for all $0<R_1<R_2<r_0$

$$\begin{aligned}&W_\lambda (R_2r_k,v)+C(R_2r_k)^{k+\gamma -\lambda }-W_ \lambda (R_1r_k,v)-C(R_1r_k)^{k+\gamma -\lambda }\\ {}&\quad \ge \int _{R_1r_k}^{R_2r_k}\frac{2}{r^{n+a+2\lambda +1}}\int _{\partial B_r(x_0)}(\nabla v\cdot x -\lambda v)^2|y|^a\,d\mathcal {H}^n\,dr\\ {}&\quad =\int _{R_1}^{R_2}\frac{2}{r^{n+a+2\lambda +1} r_k^{n+a+2\lambda }}\int _{\partial B_{rr_k}(x_0)}(\nabla v\cdot x -\lambda v)^2|y|^a\,d\mathcal {H}^n\,dr\\ {}&\quad =\int _{R_1}^{R_2}\frac{2}{r^{n+a+2\lambda +1}}\int _{\partial B_{r}}(\nabla v_r\cdot x -\lambda v_r)^2|y|^a\,d\mathcal {H}^n\,dr, \end{aligned}$$

where sending $r_k\rightarrow 0^+$ the left-hand side vanishes, by (24). Thus, by arbitrariness of $R_1$ and $R_2$, we obtain that $v_0$ is homogeneous, since $\nabla v_0\cdot x=\lambda v_0$ on $\partial B_r$ for all $r\in (0,r_0)$. $\square$

### The operator $L_a^S$

The strategy to prove the epiperimetric inequalities is to decompose a trace $c\in H^1(\partial B_1,a)$ in terms of the eigenfunctions of the operator $L_a$ restricted to $\partial B_1$. The restriction $L_a^S$ is defined for any function $\phi \in H^1(\partial B_1,a)$ as

28 $$\begin{aligned} L_a^S\phi =|y|^{-a}\text{ div }(|y|^a\nabla \Phi )|_{|x |=1}, \end{aligned}$$

where $$\Phi (x)=\phi \left( \frac{x}{|x |}\right) $$.

#### Remark 2.10

Since in spherical coordinates we have

29 $$\begin{aligned} \text{ div }(|y|^a\nabla u)= |y|^a\frac{\partial ^2 u}{\partial r^2}+\frac{1}{r}(n+a)|y|^a\frac{\partial u}{\partial r}+\frac{1}{r^2}|y|^aL_a^S u, \end{aligned}$$

then

$$\begin{aligned} L_a^S(r^\alpha \phi (\theta ))=0 \end{aligned}$$

if and only if

$$\begin{aligned} -L_a^S\phi (\theta )=\lambda ^a(\alpha )\phi (\theta ), \end{aligned}$$

where $$\lambda ^a(\alpha )=\alpha (\alpha +n+a-1)$$.

By Liouville Theorem 9.5, if we suppose that $\phi$ is even in the *y* direction, then $r^\alpha \phi (\theta )$ is $L_a$-harmonic if and only if $r^\alpha \phi (\theta )$ is a polynomial $L_a$-harmonic with $\alpha \in \mathbb {N}$.

Using the theory of compact operators, we can prove that there exist an increasing sequence of eigenvalues $\{\lambda ^a_k\}_{k\in \mathbb {N}}\subset \mathbb {R}_{\ge 0}$ and a sequence of eigenfunctions $\{\phi _k\}_{k\in \mathbb {N}}\subset H^1(\partial B_1,a)$ normalized in $L^2(\partial B_1,a)$, such that

$$\begin{aligned} -L_a^S\phi _k=\lambda ^a_k \phi _k, \end{aligned}$$

with $\{\phi _k\}_{k\in \mathbb {N}}$ orthonormal basis of $H^1(\partial B_1,a)$.

The (normalized) eigenspace corresponding to eigenvalue $\lambda ^a$ is

$$\begin{aligned} E(\lambda ^a)=\{\phi \in H^1(\partial B_1,a): -L_a^S \phi =\lambda ^a \phi , \ \Vert \phi \Vert _{L^2({\partial B_1,a})}=1\}. \end{aligned}$$

for all $$\lambda ^a\subset \{\lambda _k^a\}_{k\in \mathbb {N}}$$.

We denote by $\alpha _k\in \mathbb {N}$ the grade of the polynomial that corresponds to the eigenvalue $\lambda ^a_k$, i.e. the only natural number such that $\lambda ^a(\alpha _k)=\lambda ^a_k$.

In particular $\lambda ^a_1=\lambda ^a(0)=0$ and $E(\lambda ^a_1)$ is the space of constant functions, while $\lambda ^a_2=\ldots =\lambda ^a_{n+2}=\lambda ^a(1)=n+a$ and $E(\lambda ^a_2)$ is the space of linear functions. Finally $\lambda ^a_k \ge \lambda ^a(2)$ for $k\ge n+3$.

### The Weiss’ energy *W* and eigenfunctions of $-L_a^s$

The following lemma is a generalization of Lemma 2.3 and Lemma 2.4 in [8], for the Weiss’ energy *W* with weight. It will be used in several proof later.

#### Lemma 2.11

Let $$\phi \in H^1(\partial B_1,a)$$ with

$$\begin{aligned} \phi (\theta )=\sum _{k=1}^{\infty } c_k \phi _k(\theta )\in H^1(\partial B_1,a), \end{aligned}$$

where $\phi _k$ normalized eigenfunctions of $-L_a^S$ as above, and let $r^{\mu }\phi (\theta )$ be the $\mu$-homogeneous extension, then

30 $$\begin{aligned} W_\mu (r^\mu \phi )=\frac{1}{n+a+2\mu -1}\sum _{k=1}^{\infty } \left( \lambda _k^a-\lambda ^a(\mu )\right) c_k^2. \end{aligned}$$

Moreover, if

31 $$\begin{aligned} \kappa _{\alpha ,\mu }^a=\frac{\alpha -\mu }{\alpha +\mu +n+a-1}, \end{aligned}$$

then

32 $$\begin{aligned} W_\mu (r^\alpha \phi )-(1\mp \kappa _{\alpha ,\mu }^a)W_\mu (r^\mu \phi ) =\frac{\pm \kappa _{\alpha ,\mu }^a}{n+a+2\alpha -1}\sum _{k=1}^{\infty } (\lambda ^a(\alpha )-\lambda ^a_k)c_k^2. \end{aligned}$$

Finally, if $c\in H^1(\partial B_1)$ such that $r^{\mu +t}c$ is a solution of (4) (with 0 obstacle), then

33 $$\begin{aligned} W_\mu (r^{\mu +t}c)=t\Vert c\Vert _{L^2(\partial B_1,a)}^2 \end{aligned}$$

and

34 $$\begin{aligned} W_\mu (r^{\mu }c)=\left( 1+\frac{t}{n+a+2\mu -1}\right) W_\mu (r^{\mu +t}c). \end{aligned}$$

#### Proof

The proof is very similar to the one in [8], but we briefly recall it for the sake of completeness.

By (65) we get

$$\begin{aligned}\begin{aligned} W_\mu (r^\alpha \phi )=\sum _{k=1}^\infty c_k^2\left( \frac{\lambda _k^a+\alpha ^2}{n+a+2\alpha -1}-\mu \right) , \end{aligned} \end{aligned}$$

from where (30) and (32) follow.

Now if $r^{\mu +t}c$ is a solution, then $W_{\mu +t}(r^{\mu +t}c)=0$, by (68). Therefore (33) holds.

Finally, the proof of (34) follows by (66), (67) and (33). $\square$

### Properties of $h_e^s$

Finally we recall the properties of the function $h_e^s$ defined in (12), which is the only $(1+s)$-homogeneous solution of (4) (with 0 obstacle). The latter follows from Proposition 1.4, but it was originally proved in [7].

#### Proposition 2.12

Let $e\in \partial B_1'$ and $h_e^s$ as in (12), then $h_e^s$ is a $(1+s)$-homogeneous solution of (4) (with 0 obstacle), $h_e^s=0$ on $B'_1\cap \{x\cdot e\le 0\}$ and it holds

$$\begin{aligned} \lim _{y\rightarrow 0^+}(|y|^a\partial _yh_e^s)= {\left\{ \begin{array}{ll} -c_s|x\cdot e |^{1-s} &{} B'_1\cap \{x\cdot e <0\}\\ 0 &{} B'_1\cap \{x\cdot e \ge 0\}, \end{array}\right. } \end{aligned}$$

with $c_s=2^{1-s}(1+s)$. Finally, the $L^2(\partial B_1,a)$ projection on linear functions of $h_e^s$ has the form $C(x\cdot e)$ for some $C>0$.

#### Proof

The proof is a simple computation, hence it will be omitted. $\square$

## Epiperimetric inequality for $W_{1+s}$

The proof of the epiperimetric inequality for $W_{1+s}$ follows the ideas of the proof from [8] in the case $s=\frac{1}{2}$, i.e. we decompose the trace $c\in H^1(\partial B_1,a)$ in terms of eigenfunction of $L_a^S$.

### Decomposition of *c*

Let $c\in H^1(\partial B_1,a)$ even in the *y* direction and such that $c\ge 0$ in $\partial B'_1$. We decompose *c* using eigenfunctions of the operator $-L_a^S$ defined in (28).

The projection on linear functions $E(\lambda _2^a)$ of *c* has the form $c_1(x\cdot e)$ for some $e\in \partial B'_1$, then the projection of $h_e^s$ on $E(\lambda _2^a)$ has the same form $C(x\cdot e)$ for $C>0$. Thus we can choose $C>0$ such that *c* and $Ch_e^s$ has the same projection on $E(\lambda _2^a)$.

Notice that the function $u_0(x,y)=|y |^{1+s}$ restricted on $\partial B_1$ has 0 projection on $E(\lambda _2^a)$. Therefore we can choose $c_0\in \mathbb {R}$ such that the projections of $c-Ch_e^s$ and $u_0$ on the constant functions $E(\lambda ^a_1)$ are the same. Then

$$\begin{aligned} c(\theta )=Ch_e^s(\theta )+c_0u_0(\theta )+\phi (\theta ), \end{aligned}$$

where

35 $$\begin{aligned} \phi (\theta )=\sum _{\{k:\ \lambda ^a_k\ge \lambda ^a(2)\}} c_k\phi _k(\theta ). \end{aligned}$$

Hence we can decompose *z* as

$$\begin{aligned} z(r,\theta )=Cr^{1+s}h_e^s(\theta )+c_0r^{1 +s}u_0(\theta )+r^{1+s}\phi (\theta ) \end{aligned}$$

and we can define the competitor $\zeta$ as

$$\begin{aligned} \zeta (r,\theta )=Cr^{1+s}h_e^s(\theta )+c_0r^{ 1+s}u_0(\theta )+r^{2}\phi (\theta ), \end{aligned}$$

which is an admissible function ($\zeta \in \mathcal {K}_c$), since $\zeta (r,\theta )\ge r^2c(\theta )$ on $B'_1$.

### Proof of Theorem 1.1

Let’s start with a lemma, that will allow us to compute the Weiss’ energy $W_{1+s}$ of *z* and $\zeta$.

#### Lemma 3.1

If $\psi =r^\alpha \phi (\theta )$, with $\phi \in H^1(\partial B_1,a)$, then

$$\begin{aligned} \begin{aligned} W_{1+s}(Ch_e^s+cu_0+\psi )&=-c_0^2(1+s)(1-s)\int _{B_1}|y|\,dX+W_{1+s}(\psi )\\ {}&\quad +\frac{1}{n+\alpha +1-s}\beta (\phi ), \end{aligned} \end{aligned}$$

where

$$\begin{aligned} \begin{aligned} \beta (\phi )&:=-2c_0(1+s)(1-s)\int _{\partial B_1} \phi (\theta )|\theta _{n+1}|^{-s}\,d\mathcal {H}^{n}\\ {}&\quad +4c_sC\int _{\partial B'_1} \phi (\theta ')(\theta '\cdot e)_-^{1-s}\,d\mathcal {H}^{n-1}. \end{aligned} \end{aligned}$$

#### Proof

By Proposition 2.12 and (68), we obtain

$$\begin{aligned}&W_{1+s}(Ch_e^s+c_0u_0+\psi )=C^2W_{1+s}(h_e^s)+W_{1+s}(c_0u_0+\psi )\\&\quad +2C\biggl (\int _{B_1}\nabla h_e^s\cdot \nabla (c_0u_0+\psi )|y|^a\,dX\\ {}&\quad -(1+s)\int _{\partial B_1}h_e^s(c_0u_0+\psi )|y|^a\,d\mathcal {H}^n \biggl )\\ {}&=W_{1+s}(c_0u_0+\psi )+2C\left( \int _{B_1}\nabla h_e^s\cdot \nabla \psi |y|^a\,dX-({1+s})\int _{\partial B_1}h_e^s\psi |y|^a\,d\mathcal {H}^n\right) \\&=W_{1+s}(c_0u_0+\psi )+4c_sC\left( \int _{B'_1}\psi (x,0) (x\cdot e)_-^{1-s}\,d\mathcal {H}^n\right) \\&=W_{1+s}(cu_0+\psi )+4c_sC \int _0^1\int _{\partial B'_1} r^\alpha \phi (\theta ')r^{1-s}(\theta '\cdot e)_-^{1-s}r^{n-1}\,d\mathcal {H}^{n-1}(\theta ')\,dr\\&=W_{1+s}(c_0u_0+\psi )+\frac{4c_sC}{n+\alpha +1-s}\int _{\partial B'_1} \phi (\theta ')(\theta '\cdot e)_-^{1-s}\,d\mathcal {H}^{n-1}(\theta ') \end{aligned}$$

Additionally, since $u_0$ is $(1+s)$-homogeneous and

$$\begin{aligned} L_au_0|y|^a=(1+s)(1-s)|y|^{-s}, \end{aligned}$$

we have that

$$\begin{aligned} W_{1+s}(cu_0+\psi )&=c_0^2W_{1+s}(u_0)+W_{1+s}(\psi )\\&\quad +2c_0\left( \int _{B_1}\nabla u_0\cdot \nabla \psi |y|^a\,dX-({1+s})\int _{\partial B_1}u_0\psi |y|^a\,d\mathcal {H}^n\right) \\&=c_0^2W_{1+s}(u_0)+W_{1+s}(\psi )-2c_0\int _{B_1}\psi L_a u_0|y|^a\,dX\\ {}&=c_0^2W_{1+s}(u_0)+W_{1+s}(\psi )-2c_0(1+s)(1-s)\int _{B_1}\psi |y |^{-s}\,dX \\ {}&=c_0^2W_{1+s}(u_0)+W_{1+s}(\psi )\\&\quad -2c_0(1-s)({1+s})\int _0^1\int _{\partial B_1} r^\alpha \phi (\theta )r^{-s}|\theta _{n+1}|^{-s}r^{n}\,d\mathcal {H}^{n}(\theta )\,dr\\&=c_0^2W_{1+s}(u_0)+W_{1+s}(\psi )\\ {}&\quad -\frac{2c_0(1+s)(1-s)}{n+\alpha +1-s}\int _{\partial B_1} \phi (\theta )|\theta _{n+1}|^{-s}\,d\mathcal {H}^{n}(\theta ). \end{aligned}$$

Finally, we notice that

36 $$\begin{aligned} W_{1+s}(u_0)=-\int _{B_1}u_0L_a u_0|y|^a\,dX=-(1+s)(1-s)\int _{B_1}|y|^{1+s}|y|^{-s}\,dX<0, \end{aligned}$$

which, together with the previous identities, gives the claim. $\square$

#### Proof of Theorem 1.1

By this lemma, we can easily conclude the proof of the epiperimetric inequality for $W_{1+s}$. Indeed, if $\kappa =\kappa ^a_{2,1+s}=\frac{1+a}{2n+a+5}$, we can use

$$\begin{aligned} \frac{1}{n+2+1-s}-\left( 1-\frac{1+a}{2n+a+5}\right) \left( \frac{1}{n+(1+s)+1-s}\right) =0, \end{aligned}$$

with (32), (35) and (36) to conclude. $\square$

#### Remark 3.2

If the equality in the epiperimetric inequality holds, then $c_0=0$ and, by (32), $\phi$ is an eigenfunction corresponding to the eigenvalue $\lambda ^a(2)$. Therefore

$$\begin{aligned} z(r,\theta )=Cr^{1+s}h_e^s(\theta )+r^{1+s}\phi (\theta ). \end{aligned}$$

Furthermore, since $z\ge 0$ on $B'_1$ and $h_e^s=0$ on $B'_1\cap \{x\cdot e<0\}$, we have that $r^{1+s}\phi \ge 0$ on $B'_1\cap \{x\cdot e<0\}$, but $r^{1+s}\phi$ is even in the *y* direction, so $r^{1+s}\phi \ge 0$ on $B'_1$.

## Logarithmic epiperimetric inequality for $W_{2m}$

The proof of the logarithmic epiperimetric inequality for $W_{2m}$ follows the ideas of the proof from [8] in the case $s=\frac{1}{2}$. The strategy is the same as the one of the proof of Theorem 1.1.

### Construction of $h_{2m}$

For the proof of the logarithmic epiperimetric inequality, we need to build an eigenfunction of $-L_a^S$ as follows.

#### Remark 4.1

There is a 2*m*-homogeneous $L_a$-harmonic polynomial $h_{2m}$ such that $h_{2m}\equiv 1$ on $\partial B'_1$.

The polynomial is given by

$$\begin{aligned} h_{2m}=\sum _{k=0}^m C_k y^{2k}(x_1^2+\ldots +x_n^2)^{m-k}, \end{aligned}$$

where the constants $C_k$ are yet to be chosen.

Notice that

$$\begin{aligned} \begin{aligned}\sum _{i=1}^{n}\partial _{i,i} h_{2m}&=\sum _{k=0}^{m-1}4C_k(m-k)(m-k-1)y^{2k}(x_1^2+\ldots +x_n^2)^{m-k-1}\\&\quad +\sum _{k=0}^{m-1}2nC_k(m-k)y^{2k}(x_1^2+\ldots +x_n^2)^{m-k-1}\\&=\sum _{k=1}^{m}C_{k-1}y^{2k-2}2(m-k+1)(2(m-k)+n)(x_1^2+\ldots +x_n^2)^{m-k},\\ \partial _{y,y} h_{2m}&=\sum _{k=1}^{m}C_k(2k)(2k-1)y^{2k-2}(x_1^2+\ldots +x_n^2)^{m-k} \end{aligned} \\ \end{aligned}$$

and

$$\begin{aligned} \frac{a}{y}\partial _y h_{2m} =\sum _{k=1}^maC_k(2k)y^{2k-2}(x_1+\ldots x_n)^{m-k}. \end{aligned}$$

Thus, $h_{2m}$ is $L_a$-harmonic if and only if

$$\begin{aligned} C_k(2k)(2k-1+a)+2(m-k+1)(2(m-k)+n)C_{k-1}=0. \end{aligned}$$

Therefore, we can choose

$$\begin{aligned} C_{k}=-\frac{2(m-k+1)(2(m-k)+n)}{2k(2k-1+a)}C_{k-1}, \end{aligned}$$

for $k\in \{1,\ldots m\}$ and $C_0=1$, which concludes the construction of $h_{2m}$.

### Decomposition of *c*

Let $c\in H^1(\partial B_1,a)$, we can decompose

$$\begin{aligned} c(\theta )=\sum _{k=1}^\infty c_k \phi _k(\theta ), \end{aligned}$$

where $\phi _k(\theta )$ are the normalized eigenfunctions of $-L_a^S$ with eigenvalues $\lambda _k^a$ and corresponding homogeneity $\alpha _k$, then

$$\begin{aligned} c(\theta )=P(\theta )+\phi (\theta ), \end{aligned}$$

with

$$\begin{aligned} P(\theta )=\sum _{\{k:\ \alpha _k\le 2m\}}c_k \phi _k(\theta ) \end{aligned}$$

and

$$\begin{aligned} \phi (\theta )=\sum _{\{k:\ \alpha _k>2m\}}c_k \phi _k(\theta ). \end{aligned}$$

Let $z=r^{2m}c$ be the 2*m*-homogeneous extension of *c* and let $h_{2m}$ as in Remark 4.1, therefore

$$\begin{aligned} z(r,\theta )=r^{2m}P(\theta )+Mr^{2m}h_{2m}(\theta ) -Mr^{2m}h_{2m}(\theta )+r^{2m}\phi (\theta ), \end{aligned}$$

where $M=\max \{P_-(\theta '):\theta '\in \partial B'_1\}$ and $P_-(\theta )$ is the negative part of $P(\theta )$.

We choose a competitor $\zeta$ extending with homogeneity $\alpha >2m$ the high modes on the sphere and leaving the rest unchanged, i.e.

$$\begin{aligned} \zeta (r,\theta )=r^{2m}P(\theta )+Mr^{2m}h_{2m}(\theta ) -Mr^{\alpha }h_{2m}(\theta )+r^{\alpha }\phi (\theta ), \end{aligned}$$

for some $2m<\alpha <2m+\frac{1}{2}$, then

$$\begin{aligned} \zeta (r,\theta )\ge r^\alpha c(\theta )\ge 0 \end{aligned}$$

on $B'_1$, since we have chosen $M>0$ such that $P(\theta )+M\ge 0$ $\forall \theta \in \partial B'_1$. This means that $\zeta \in \mathcal {K}_c$.

Defining $\kappa _{\alpha ,2m}^a$ as in (31), we will choose $\varepsilon =\varepsilon (n,m)>0$ small enough and $2m<\alpha <2m+\frac{1}{2}$ such that

37 $$\begin{aligned} \kappa _{\alpha ,2m}^a=\varepsilon \Theta ^{-\beta } \Vert \nabla _\theta \phi \Vert _{L^2(\partial B_1,a)}^{2\beta } \end{aligned}$$

for $\Theta >0$ and $\beta =\frac{n-1}{n+1}$. Notice that to be able to choose such $\alpha$, we must have an estimate of the type

38 $$\begin{aligned} \Vert \nabla _\theta \phi \Vert _{L^2(\partial B_1,a)}^{2}\le C_{n,m,a}\Theta , \end{aligned}$$

for some $C_{n,m}>0$, that should depend only on *n*, *m* and *a*, since we want that $\alpha$ depends only on *n*, *m* and *a*. For this reason we ask for the bounds $\Vert c\Vert _{L^2(\partial B_1),a}^2\le \Theta$ and $|W_{2\,m}(z)|\le \Theta$.

### Proof of Theorem 1.2

First we want to compute the term $W_{2m}(\zeta )-(1-\kappa _{\alpha ,2m}^a)W_{2m}(z)$, and we see that for $\alpha$ near $2\,m$ and $\alpha >2\,m$, it is negative. This is contained in the following lemmas.

#### Lemma 4.2

In the hypotheses of Theorem 1.2, we have

39 $$\begin{aligned} W_{2m}(\zeta )-(1-\kappa _{\alpha ,2m}^a)W_{2m}(z)\le C_1M^2 (\kappa ^a_{\alpha ,2m})^2-C_2\kappa ^a_{\alpha ,2m} \Vert \nabla _\theta \phi \Vert _{L^2(\partial B_1,a)}^{2}, \end{aligned}$$

for some $$ C_1,C_2>0$$ depending only on *n*, *m* and *a*.

#### Proof

We introduce the following functions:

$$\begin{aligned}{} & {} \psi (r,\theta )=\sum _{\{k:\ \alpha _k<2m\}} c_k r^{2m} \phi _k(\theta ), \\{} & {} H_{2m}(r,\theta )=Mr^{2m}h_{2m}(\theta )+\sum _{\{k:\ \alpha _k=2m\}} c_k r^{2m} \phi _k(\theta ) \end{aligned}$$

and

$$\begin{aligned} \varphi _\mu (r,\theta )=-Mr^\mu h_{2m}(\theta )+\sum _{\{k:\ \alpha _k>2m\}} c_k r^{\mu } \phi _k(\theta ). \end{aligned}$$

Then, we can write $z=\psi +H_{2m}+\varphi _{2m}$ and $\zeta =\psi +H_{2m}+\varphi _\alpha$.

With this decomposition, $\psi$ is orthogonal in $L^2(B_1,a)$ and in $H^1(B_1,a)$ to $\varphi _\mu$ for $\mu =\alpha ,2m$. Additionally, $H_{2m}$ is $L_a$-harmonic and 2*m*-homogeneous, therefore using (68), we get

$$\begin{aligned} W_{2m}(z)=W_{2m}(\psi +\varphi _{2m}) =W_{2m}(\psi )+W_{2m}(\varphi _{2m}) \end{aligned}$$

and

$$\begin{aligned} W_{2m}(\zeta )=W_{2m}(\psi +\varphi _{\alpha }) =W_{2m}(\psi )+W_{2m}(\varphi _{\alpha }). \end{aligned}$$

Notice now that, since $\psi$ has only frequencies lower than 2*m*, we have $W_{2m}(\psi )<0$. Thus, using (30), we get

$$\begin{aligned} \begin{aligned} W_{2m}(\zeta )-(1-&\kappa ^a_{\alpha ,2m})W_{2m}(z)\\&=\kappa ^a_{\alpha ,2m}W_{2m}(\psi )+W_{2m}(\varphi _\alpha ) -(1-\kappa ^a_{\alpha ,2m})W_{2m}(\varphi _{2m})\\&\le W_{2m}(\varphi _\alpha )-(1-\kappa ^a_{\alpha ,2m})W_{2m}(\varphi _{2m})\\&=M^2\Vert h_{2m}\Vert _{L^2(\partial B_1,a)}^2 \frac{\kappa ^a_{\alpha ,2m}}{n+a+2\alpha -1}(\lambda ^a(\alpha )-\lambda ^a(2m))\\&\quad -\frac{\kappa ^a_{\alpha ,2m}}{n+a+2\alpha -1}\sum _{\{k:\ \alpha _k>2m\}}(\lambda _k^a-\lambda ^a(\alpha ))c_k^2\\&=M^2\Vert h_{2m}\Vert _{L^2(\partial B_1,a)}^2(\kappa ^a_{\alpha ,2m})^2\frac{(\alpha +2m+n+a-1)^2}{n+a+2\alpha -1}\\&\quad -\frac{\kappa ^a_{\alpha ,2m}}{n+a+2\alpha -1}\sum _{\{k:\ \alpha _k>2m\}}(\lambda _k-\lambda (\alpha ))c_k^2\\&\le C_1M^2(\kappa ^a_{\alpha ,2m})^2-\overline{C}\kappa ^a_{\alpha ,2m}\sum _{\{k:\ \alpha _k>2m\}}(\lambda _k-\lambda (\alpha ))c_k^2, \end{aligned} \end{aligned}$$

where in the second equality we used (32) with $C_1$ and ${\overline{C}}$ depending only on *n*, *m* and *a*.

Observe that

40 $$\begin{aligned} \begin{aligned} \sum _{\{k:\ \alpha _k>2m\}} (\lambda ^a_k-\lambda ^a(\alpha ))c_k^2&=\sum _{\{k:\ \alpha _k>2m\}} \lambda ^a_kc_k^2-\lambda ^a(\alpha )\sum _{\{k:\ \alpha _k>2m\}} c_k^2\\&\ge \sum _{\{k:\ \alpha _k>2m\}} \lambda ^a_kc_k^2-\frac{\lambda ^a(\alpha )}{\lambda ^a(2m+1)}\sum _{\{k:\ \alpha _k>2m\}} \lambda ^a_kc_k^2\\&\ge \sum _{\{k:\ \alpha _k>2m\}} \lambda ^a_kc_k^2-\frac{\lambda ^a(2m+\frac{1}{2})}{\lambda ^a(2m+1)}\sum _{\{k:\ \alpha _k>2m\}} \lambda ^a_kc_k^2\\&\ge {\overline{C}}_2\sum _{\{k:\ \alpha _k>2m\}}\lambda ^a_kc_k^2={\overline{C}}_2\Vert \nabla _\theta \phi \Vert _{L^2(\partial B_1,a)}^{2}, \end{aligned} \end{aligned}$$

where we used

$$\begin{aligned} \Vert \nabla _\theta \phi _k\Vert ^2_{L^2(\partial B_1,a)}=\lambda ^a_k\Vert \phi _k\Vert ^2_{L^2(\partial B_1,a)}\end{aligned}$$

and we have chosen

$$\begin{aligned} {\overline{C}}_2=\frac{\lambda ^a(2m+1)-\lambda ^a (2m+\frac{1}{2})}{\lambda ^a(2m+1)}. \end{aligned}$$

Hence, we conclude by choosing $C_2={\overline{C}} {\overline{C}}_2$ and $C_1$ as above. $\square$

#### Lemma 4.3

In the same hypotheses of the Theorem 1.2, we have

41 $$\begin{aligned} M^2\le C_3\Theta ^\beta \Vert \nabla _\theta \phi \Vert _{L^2(\partial B_1,a)}^{2(1-\beta )}, \end{aligned}$$

for some $C_3>0$ that depends only on *n*, *m* and *a*.

#### Proof

Since $\phi _k$ are $C^1$ on $\partial {B_1}$ and only a finite number of *k* is such that $\alpha _k\le 2m$, we have

$$\begin{aligned} \Vert \nabla _\theta \phi _k\Vert _{L^\infty (\partial B_1)}^{2}\le L_m \end{aligned}$$

for all *k* such that $\alpha _k\le 2m$ and for some $L_m>0$.

Moreover, the coefficients $c_k$ corresponding to *P* are bounded by $\Theta ^\frac{1}{2}$, since $\Vert P\Vert _{L^2(\partial B_1,a)}^2\le \Vert c\Vert _{L^2(\partial B_1,a)}^2\le \Theta$, we get *P* is *L*-Lipschitz continuous on $\partial {B_1}$, with $L=L_{n,m,a}\Theta ^\frac{1}{2}$.

Now, since $\phi ^2(\theta )\ge P_-^2(\theta )$ for all $\theta \in \partial B'_1$, it follows that

$$\begin{aligned} \begin{aligned} \int _{\partial B'_1}P_-^2\,d\mathcal {H}^{n-1}&\le \int _{\partial B'_1} \phi ^2\,d\mathcal {H}^{n-1}\\&\le C\left( \int _{\partial B_1} |\nabla _\theta \phi |^2|y|^a\,d\mathcal {H}^n+\int _{ \partial B_1} \phi ^2|y|^a\,d\mathcal {H}^n\right) \\&\le C\left( \int _{\partial B_1} |\nabla _\theta \phi |^2|y|^a\,d\mathcal {H}^n+\sum _{\{k: \ \alpha _k>2m\}}\frac{1}{\lambda ^a_k}\int _{ \partial B_1} |\nabla _\theta \phi _k|^2|y|^a\,d\mathcal {H}^n\right) \\&\le C\left( 1+\frac{1}{\lambda ^a(2m)}\right) \int _{ \partial B_1} |\nabla _\theta \phi |^2|y|^a\,d\mathcal {H}^n, \end{aligned} \end{aligned}$$

by Proposition 9.6, and since $\phi$ contains only eigenfunctions corresponding to eigenvalue $\lambda ^a_k>\lambda ^a(2m)$.

Finally we claim that

$$\begin{aligned} \int _{\partial B_1'}P_-^2(\theta )\,d\mathcal {H}^{n-1}\ge C M^2 \left( \frac{M}{L}\right) ^{n-1}=C\frac{{M}^{n+1}}{{L}^{n-1}}, \end{aligned}$$

in fact the norm in $L^2$ of $P_-^2(\theta )$ is controlled by the volume of an $(n-1)$-dimensional cone with height $M^2$ and radius of the base $\frac{M}{L}$, since the graph of $P_-$ must be above this cone, by the Lipschitz continuity of $P_-$.

Thus, since $1-\beta =\frac{2}{n+1}$ and $L=L_{n,m}\Theta ^\frac{1}{2}$, we obtain

$$\begin{aligned} M^2\le C L^{2\frac{n-1}{n+1}}\Vert \nabla _\theta \phi \Vert _{L^2(\partial B_1,a)}^{\frac{4}{n+1}}=C_3\Theta ^\beta \Vert \nabla _\theta \phi \Vert _{L^2(\partial B_1,a)}^{2(1-\beta )}, \end{aligned}$$

which is precisely (41). $\square$

#### Proof of Theorem 1.2

Notice that we can suppose $W_{2m}(z)>0$, otherwise we have done.

First, as already observed, we must show the estimate (38).

In fact, as in (40) with $\alpha =2m$, we have

$$\begin{aligned} \Vert \nabla _\theta \phi \Vert _{L^2(\partial B_1,a)}^{2}\le C_{n,m,a}\sum _{\{k:\ \alpha _k>2m\}} (\lambda _k-\lambda (2m))c_k^2=C_{n,m,a}W_{2m}(r^{2m}\phi ), \end{aligned}$$

where in the last equality we used (30).

Using the orthogonality of *P* and $\phi$ and again (30), we obtain

$$\begin{aligned} \begin{aligned}W_{2m}(r^{2m}\phi )&=W_{2m}(z)-W_{2m}(r^{2m}P(\theta ))\\&\le W_{2m}(z)+\frac{\lambda ^a(2m)}{n+a+4m-1}\Vert P\Vert _{L^2(\partial B_1,a)}^2\\ {}&\le W_{2m}(z)+2m\Vert P\Vert _{L^2(\partial B_1,a)}^2\le W_{2m}(z)+2m\Vert c\Vert _{L^2(\partial B_1,a)}^2\\ {}&\le (1+2m)\Theta , \end{aligned} \end{aligned}$$

where we used that $\Vert c\Vert _{L^2(\partial B_1)}^2\le \Theta$ and $|W_{2m}(z)|\le \Theta$, that concludes the estimate.

If we choose $\varepsilon <\frac{C_2}{C_1C_3}$, where $C_1,C_2,C_3$ are the constant in (39) and (41) and if we choose $\kappa _{\alpha ,2m}$ as in (37), we deduce that

42 $$\begin{aligned} \begin{aligned}&W_{2m}(\zeta )-(1-\kappa _{\alpha ,2m})W_{2m}(z)\le C_1M^2 \kappa _{\alpha ,2m}^2-C_2\kappa _{\alpha ,2m} \Vert \nabla _\theta \phi \Vert _{L^2(\partial B_1,a)}^{2}\\ {}&\quad \le C_1C_3 \kappa _{\alpha ,2m}^2\Theta ^\beta \Vert \nabla _\theta \phi \Vert _{L^2(\partial B_1,a)}^{2(1-\beta )}-C_2\kappa _{\alpha ,2m} \Vert \nabla _\theta \phi \Vert _{L^2(\partial B_1,a)}^{2}\\ {}&\quad =C_1C_3 \varepsilon ^2\Theta ^{-\beta }\Vert \nabla _\theta \phi \Vert _{L^2(\partial B_1,a)}^{4\beta }\Vert \nabla _\theta \phi \Vert _{L^2(\partial B_1,a)}^{2(1-\beta )}\\ {}&\qquad -C_2\varepsilon \Theta ^{-\beta }\Vert \nabla _\theta \phi \Vert _{L^2(\partial B_1,a)}^{2\beta } \Vert \nabla _\theta \phi \Vert _{L^2(\partial B_1,a)}^{2}\\ {}&\quad =\varepsilon (\varepsilon C_1C_3-C_2)\Theta ^{-\beta }\Vert \nabla _\theta \phi \Vert _{L^2(\partial B_1,a)}^{2+2\beta }<0. \end{aligned} \end{aligned}$$

Finally

$$\begin{aligned} \begin{aligned} W_{2m}(z)&=\frac{1}{n+a+4m-1}\sum _{k=1}^\infty (\lambda ^a_k-\lambda ^a(2m))c_k^2\\&\le \frac{1}{n+a+4m-1}\sum _{\{k:\ \alpha _k>2m\}}(\lambda _k^a-\lambda ^a(2m))c_k^2\\&\le \sum _{\{k:\ \alpha _k>2m\}}\lambda ^a_kc_k^2=\Vert \nabla _\theta \phi \Vert _{L^2(\partial B_1,a)}^{2}, \end{aligned} \end{aligned}$$

therefore

$$\begin{aligned} \begin{aligned} W_{2m}(\zeta )&\le (1-\kappa _{\alpha ,2m})W_{2m}(z)=(1-\varepsilon \Theta ^{-\beta } \Vert \nabla _\theta \phi \Vert _{L^2(\partial B_1,a)}^{2\beta })W_{2m}(z)\\&\le (1-\varepsilon \Theta ^{-\beta } W_{2m}(z)^\beta )W_{2m}(z),\end{aligned} \end{aligned}$$

that conclude the proof. $\square$

#### Remark 4.4

We have proved a stronger version of the logarithmic epiperimetric inequality, that is

43 $$\begin{aligned} W_{2m}(\zeta )\le W_{2m}(z)(1-\varepsilon \Theta ^{-\beta }|W_{2m}(z)|^\beta )-\varepsilon _1\Theta ^{-\beta }\Vert \nabla _\theta \phi \Vert _{L^2(\partial B_1,a)}^{2+2\beta }, \end{aligned}$$

for $\varepsilon _1=\frac{C_2\varepsilon }{2}$, choosing $\varepsilon <\frac{C_2}{2C_1C_3}$ in (42).

## Epiperimetric inequalities for negative energies

In this section we prove two epiperimetric inequalities for negative energies^2^$W_{1+s}$ and $W_{2m}$.

These epiperimetric inequalities are a generalization for the case $s=\frac{1}{2}$ and they allow us to prove the backward frequency gap in Proposition 1.3.

### Epiperimetric inequality for negative energies $W_{1+s}$

In the case $s=\frac{1}{2}$, the epiperimetric inequality for negative energies $W_{1+s}$ was proved in [5]. We follow the same idea.

#### Theorem 5.1

(Epiperimetric inequality for negative energies $W_{1+s}$) Let $\mathcal {K}_c$ be defined as in (10) and $z=r^{1+s}c(\theta )\in \mathcal {K}_c$ be the $(1+s)$-homogeneous extension in $\mathbb {R}^{n+1}$ of a function $c\in H^1(\partial B_1,a)$. Then, there is $\zeta \in \mathcal {K}_c$ such that

$$\begin{aligned} W_{1+s}(\zeta )\le (1+\varepsilon )W_{1+s}(z), \end{aligned}$$

with $$\varepsilon =\frac{1+a}{2n-a+3}.$$

#### Proof

Let *z* be the $(1+s)$-homogeneous extension of its trace $c\in H^1(\partial B_1,a)$. Then, we can decompose *z* as

$$\begin{aligned} z(r,\theta )=Cr^{1+s}h^s_e(\theta )+c_0r^{1+s}u_0(\theta )+r^{1+s}\phi (\theta ), \end{aligned}$$

with $u_0(x,y)=|y|^{2s}$, as in the proof of Theorem 1.1. Therefore the explicit competitor is

$$\begin{aligned} \zeta (r,\theta )=Cr^{1+s}h^s_e(\theta ) +c_0r^{2s}u_0(\theta )+r^{1+s}\phi (\theta ), \end{aligned}$$

which is an admissible function, since $\zeta =z\ge 0$ on $B'_1$, i.e. $\zeta \in \mathcal {K}_c$.

Now we want to compute the Weiss’ energy of $Cr^{1+s}h^s_e(\theta )+c_0r^\alpha u_0(\theta )+r^{1+s}\phi (\theta )$, for $\alpha =2s,{1+s}$. By (68), we have $W_{1+s}(h^s_e)=0$. Then

$$\begin{aligned}&W_{1+s}(Cr^{1+s}h^s_e+c_0r^\alpha u_0+r^{1+s}\phi )=C^2W_{1+s}(h^s_e)+W_{1+s}(c_0r^\alpha u_0+r^{1+s}\phi )\\ {}&\quad +2C\Biggl (\int _{B_1}\nabla (r^{1+s}h^s_e)\cdot \nabla (c_0r^\alpha u_0+r^{1+s}\phi )|y|^a\,dX\\ {}&\quad -({1+s})\int _{\partial B_1}h^s_e(c_0u_0+\phi )|y|^a\,d\mathcal {H}^n\Biggl ) \\ {}&=c_0^2W_{1+s}(r^\alpha u_0)+W_{1+s}(r^{1+s}\phi )\\ {}&\quad +2c_0\Biggl ( \int _{B_1}\nabla (r^\alpha u_0)\cdot \nabla (r^{1+s}\phi )|y|^a\,dX-({1+s})\int _{\partial B_1}u_0\phi |y|^a\,d\mathcal {H}^n\Biggl )\\ {}&\quad +2C\Biggl ( \int _{B_1}\nabla (r^{1+s}h^s_e)\cdot \nabla (r^{1+s}\phi )|y|^a\,dX-({1+s})\int _{\partial B_1}h^s_e\phi |y|^a\,d\mathcal {H}^n\Biggl ), \end{aligned}$$

where in the last equality we used that $u_0\equiv$ 0 on $B'_1$, combined with Proposition (2.12).

Integrating by parts, we get that

$$\begin{aligned} \begin{aligned}&W_{1+s}(Cr^{1+s}h^s_e+c_0r^\alpha u_0+r^{1+s}\phi )=c_0^2W_{1+s}(r^\alpha u_0)+W_{1+s}(r^{1+s}\phi )\\ {}&\qquad +2c_0\Biggl ( \int _{B_1}\nabla (r^\alpha u_0)\cdot \nabla (r^{1+s}\phi )|y|^a\,dX-({1+s})\int _{\partial B_1}u_0\phi |y|^a\,d\mathcal {H}^n\Biggl )\\ {}&\qquad +2C\Biggl (\int _{B_1} -L_a (r^{1+s}h_e^s) r^{1+s}\phi |y|^a\,dX\Biggl ). \end{aligned} \end{aligned}$$

Then

44 $$\begin{aligned} W_{1+s}(\zeta )- (1+\varepsilon )W_{1+s}(z)=I+J+K+L, \end{aligned}$$

where

$$\begin{aligned} \begin{aligned} I&=c_0^2\left( W_{1+s}(r^{2s} u_0)-(1+\varepsilon )W_{1+s}(r^{1+s} u_0)\right) , \\ J&=W_{1+s}(r^{1+s}\phi ) -(1+\varepsilon )W_{1+s}(r^{1+s}\phi ), \\ K&=2c_0\Biggl ( \int _{B_1}\nabla (r u_0)\cdot \nabla (r^{1+s}\phi )|y|^a\,dX-({1+s})\int _{\partial B_1}u_0\phi |y|^a\,d\mathcal {H}^n\\ {}&\quad -(1+\varepsilon )\Biggl (\int _{B_1}\nabla (r^{1+s} u_0)\cdot \nabla (r^{1+s}\phi )|y|^a\,dX-({1+s})\int _{\partial B_1}u_0\phi |y|^a\,d\mathcal {H}^n\Biggl )\Biggl ) \end{aligned} \end{aligned}$$

and

$$\begin{aligned} \begin{aligned}L&=2C\Biggl (\int _{B_1} -L_a (r^{1+s}h_e^s) r^{1+s}\phi |y|^a\,dX\\ {}&\quad -(1+\varepsilon )\Biggl (\int _{B_1} -L_a (r^{1+s}h_e^s) r^{1+s}\phi |y|^a\,dX\Biggl )\Biggl ). \end{aligned} \end{aligned}$$

For *I*, we notice that the function $-r^{2\,s}u_0(\theta )=-|y |^{2\,s}$ is a solution of (4) (with 0 obstacle), then using (33), we obtain

$$\begin{aligned} W_{1+s}(r^{2s}u_0)=W_{1+s}(-r^{{1+s}-(1-s)}u_0)=-(1-s)\Vert u_0\Vert _{L^2(\partial B_1,a)}^2 \end{aligned}$$

and using (34), we get

$$\begin{aligned}\begin{aligned} W_{1+s}(r^{1+s}u_0)&=\left( 1+\frac{-(1-s)}{n+2}\right) W_{1+s}(r^{2s}u_0)\\&=\left( 1+\frac{-(1-s)}{n+2}\right) \left( -(1-s)\right) \Vert u_0\Vert _{L^2(\partial B_1,a)}^2, \end{aligned} \end{aligned}$$

therefore

$$\begin{aligned} I=\left( -(1-s)-\left( 1+\varepsilon \right) \left( 1+\frac{-(1-s)}{n+2}\right) \left( -(1-s)\right) \right) \Vert u_0\Vert _{L^2(\partial B_1,a)}^2=0, \end{aligned}$$

since $\varepsilon =\frac{1+a}{2n-a+3}$, with a simple calculation.

For *J*, using (30), we deduce that

$$\begin{aligned} J=-\varepsilon W_{1+s}(r^{1+s}\phi )=-\frac{\varepsilon }{n+2}\sum _{\{k:\ \lambda ^a_k\ge \lambda ^a(2)\}}\left( \lambda ^a_k-\lambda ^a\left( {1+s}\right) \right) c_k^2\le 0, \end{aligned}$$

since $$\lambda ^a(2)\ge \lambda ^a({1+s})$$.

For *K*, we integrate by parts

$$\begin{aligned} K&=2c_0\Biggl (\int _{B_1}\nabla (r^{2s} u_0)\cdot \nabla (r^{1+s}\phi )|y|^a\,dX-2s\int _{\partial B_1}u_0\phi |y|^a\,d\mathcal {H}^n\\ {}&\quad -(1-s)\int _{\partial B_1}u_0\phi |y|^a\,d\mathcal {H}^n-(1+\varepsilon )\Biggl (\int _{B_1}\nabla (r^{1+s} u_0)\cdot \nabla (r^{1+s}\phi )|y|^a\,dX\\ {}&\quad -({1+s})\int _{\partial B_1}u_0\phi |y|^a\,d\mathcal {H}^n\Biggl )\Biggl ) \\ {}&=2c_0\Biggl ( \int _{B_1}-L_a(r^{2s} u_0)r^{1+s}\phi |y|^a\,dX-(1-s)\int _{\partial B_1}u_0\phi \,d\mathcal {H}^n\\ {}&\qquad -(1+\varepsilon )\Biggl (\int _{B_1}-L_a (r^{1+s} u_0)r^{1+s}\phi |y|^a\,dX\Biggl )\Biggl ). \end{aligned}$$

Now, by (29), we get

$$\begin{aligned} L_a (r^\alpha u_0)|y|^a=\lambda ^a(\alpha )r^{\alpha -2}u_0|y|^a+r^{\alpha -2}L_a^S u_0|y|^a, \end{aligned}$$

with $\lambda ^a(2\,s)=2ns$ and $\lambda ^a({1+s})=(1+s)n+(1+s)(1-s)$, then

$$\begin{aligned} K&=2c_0\Biggl ( \int _{B_1}-2nsr^{-2+2s}u_0r^{1+s}\phi |y|^a\,dX-\int _{B_1}r^{-2+2s}L_a^S u_0r^{1+s}\phi |y|^a\,dX\\&\quad -(1-s)\int _{\partial B_1}u_0\phi |y|^a\,d\mathcal {H}^n\\&\quad -(1+\varepsilon )\Biggl (\int _{B_1}-\left( ({1+s})n+(1+s)(1-s)\right) r^{-1+s}u_0r^{1+s}\phi \,dX\\ {}&\quad -\int _{B_1}r^{-1+s}L_a^S u_0r^{1+s}\phi |y|^a\,dX\Biggl )\Biggl )\\&=2c_0\Biggl ( -\frac{2ns}{n+{3s+a}}\int _{\partial B_1}u_0\phi |y|^a\,d\mathcal {H}^n-\frac{1}{n+{3s+a}}\int _{\partial B_1}L_a^S u_0\phi |y|^a\,d\mathcal {H}^n\\ {}&\quad -(1-s)\int _{\partial B_1}u_0\phi |y|^a\,d\mathcal {H}^n\\&\quad -(1+\varepsilon )\Biggl ( -\frac{({1+s})n+(1+s)(1-s)}{n+2}\int _{\partial B_1}u_0\phi \,d\mathcal {H}^n\\&\quad -\frac{1}{n+2}\int _{\partial B_1}L_a^S u_0\phi |y|^a\,d\mathcal {H}^n\Biggl )\Biggl ), \end{aligned}$$

where in the last equality we used (64). Hence, we obtain

$$\begin{aligned} \begin{aligned}K&=2c_0\Biggl ( -\frac{2ns}{n+{3s+a}}-(1-s)\\ {}&\quad +\left( 1+\varepsilon \right) \frac{({1+s})n+(1+s)(1-s)}{n+2}\Biggl ) \int _{\partial B_1}u_0\phi |y|^a\,d\mathcal {H}^n \\ {}&\quad +2c_0\Biggl (-\frac{1}{n+{3s+a}}+\left( 1+\varepsilon \right) \left( \frac{1}{n+2}\right) \Biggl )\int _{\partial B_1}L_a^S u_0\phi |y|^a\,d\mathcal {H}^n=0, \end{aligned} \end{aligned}$$

since $\varepsilon =\frac{1+a}{2n-a+3}$, with a simple calculation.

For *L*, by Proposition (2.12), we have

$$\begin{aligned}\begin{aligned} L&=-2C\varepsilon \left( \int _{B_1} -L_a(r^{1+s}h_e^s)r^{1+s} \phi |y|^a\,dX\right) \\ {}&=-4c_sC\varepsilon \int _{B'_1\cap \{x\cdot e<0\}}r^{1+s}\phi |x\cdot e|^{1-s}|y|^a\,d\mathcal {H}^n\le 0, \end{aligned} \end{aligned}$$

where we used that $h_e^s(\theta )=u_0(\theta )=0$ for $\theta \in B'_1\cap \{x\cdot e<0\}$ and that $\phi =c\ge 0$ in $B'_1\cap \{x\cdot e<0\}$.

Finally, since $I,J,K,L\le 0$, we conclude by using (44). $\square$

### Epiperimetric inequality for negative energies $W_{2m}$

In the case $s=\frac{1}{2}$, the epiperimetric inequality for negative energies $W_{2m}$ was proved in [8]. We follow the same idea.

#### Theorem 5.2

(Epiperimetric inequality for negative energies $W_{2m}$) Let $\mathcal {K}_c$ be defined as in (10) and let $z=r^{2m}c(\theta )\in \mathcal {K}_c$ be the 2*m*-homogeneous extension in $\mathbb {R}^{n+1}$ of a function $c\in H^1(\partial B_1,a)$ such that $\Vert c\Vert _{L^2(\partial B_1,a)}^2\le 1$. Then, there is $\zeta \in \mathcal {K}_c$ such that

$$\begin{aligned} W_{2m}(\zeta )\le (1+\varepsilon )W_{2m}(z), \end{aligned}$$

with $\varepsilon ={\varepsilon }(n,m,a)>0$ small enough.

#### Proof

Notice that we can suppose $W_{2m}(z)<0$, otherwise we have done.

We can decompose $c\in H^1(\partial B_1,a)$ as

$$\begin{aligned} c=\sum _{k=1}^\infty c_k\phi _k(\theta )={\overline{P}}(\theta ) +{\overline{\phi }}(\theta ), \end{aligned}$$

where $\phi _k(\theta )$ are as above, with

$$\begin{aligned} \overline{P}(\theta )=\sum _{\{k:\ \alpha _k<2m\}}c_k \phi _k(\theta ) \end{aligned}$$

and

$$\begin{aligned} {\overline{\phi }}(\theta )=\sum _{\{k:\ \alpha _k\ge 2m\}}c_k \phi _k(\theta ). \end{aligned}$$

We decompose $z=r^{2m}c$, the 2*m*-homogeneous extension of *c*, as

$$\begin{aligned} z(r,\theta )=r^{2m}\overline{P}(\theta )+Mr^{2m}h_{2m}(\theta )-Mr^{2m}h_{2m}(\theta )+r^{2m}\overline{\phi }(\theta ), \end{aligned}$$

where $h_{2m}$ as in Remark 4.1 and $M=\max \{P_-(\theta ): \theta \in \partial B_1'\}$ as above.

We define $\varepsilon =\kappa ^a_{2m,\alpha }$ with $\kappa ^a_{2m,\alpha }$ as in (31) and $\alpha \in (2m-\frac{1}{2},2m)$ and we will choose $\varepsilon =\varepsilon (n,m,a)>0$ small enough (which corresponds to choosing $\alpha$ close to $2\,m$ and $\alpha <2\,m$).

The explicit competitor is

$$\begin{aligned} \zeta (r,\theta )=r^{\alpha }\overline{P}(\theta )+Mr^{\alpha }h_{2m}(\theta )-Mr^{2m}h_{2m}(\theta )+r^{2m}{\overline{\phi }}(\theta ), \end{aligned}$$

where we notice that $\zeta \in \mathcal {K}_c$ since $\zeta (r,\theta )\ge r^{2m} c(\theta )\ge 0$ on $B'_1$.

As above, we can define

$$\begin{aligned} \psi _\mu (r,\theta )= & {} \sum _{\{k:\ \alpha _k<2m\}} c_k r^{\mu } \phi _k(\theta )+Mr^{\mu }h_{2m}(\theta ), \\ H_{2m}(r,\theta )= & {} -Mr^{2m} h_{2m}(\theta )+\sum _{\{k:\ \alpha _k=2m\}} c_k r^{2m} \phi _k(\theta ) \end{aligned}$$

and

$$\begin{aligned} \varphi (r,\theta )=\sum _{\{k:\ \alpha _k>2m\}} c_k r^{2m} \phi _k(\theta ), \end{aligned}$$

and we write $z=\psi _{2m}+H_{2m}+\varphi$ and $\zeta =\psi _\alpha +H_{2m}+\varphi$.

Moreover, since the functions $\psi _\mu$ are orthogonal to $\varphi$ in $L^2( B_1,a)$ and in $H^1( B_1,a)$, since $H_{2m}$ is $L_a$-harmonic and 2*m*-homogeneous, and since $W_{2m}(\varphi )>0$ by (30), we have that

$$\begin{aligned} \begin{aligned} W_{2m}(\zeta )-(1+\varepsilon )W_{2m}(z)&=W_{2m}(\zeta )-(1+\kappa ^a_{2m,\alpha })W_{2m}(z) \\ {}&=-\kappa ^a_{2m,\alpha }W_{2m}(\varphi )+W_{2m}(\psi _\alpha ) -(1+\kappa ^a_{2m,\alpha })W_{2m}(\psi _{2m})\\ {}&\le W_{2m}(\psi _\alpha )-(1+\kappa ^a_{2m,\alpha })W_{2m}(\psi _{2m})\\ {}&=M^2\Vert h_{2m}\Vert _{L^2(\partial B_1,a)}^2 \frac{\kappa ^a_{2m,\alpha }}{n+a+2\alpha -1}(\lambda ^a(2m)-\lambda ^a(\alpha ))\\&\quad -\frac{\kappa ^a_{2m,\alpha }}{n+a+2\alpha -1}\sum _{\{k:\ \alpha _k<2m\}}(\lambda ^a(\alpha )-\lambda ^a_k)c_k^2\\&=M^2\Vert h_{2m}\Vert _{L^2(\partial B_1,a)}^2(\kappa ^a_{\alpha ,2m})^2\frac{(\alpha +2m+n+a-1)^2}{n+a+2\alpha -1} \\&\quad -\frac{\kappa ^a_{\alpha ,2m}}{n+a+2\alpha -1}\sum _{\{k:\ \alpha _k<2m\}}(\lambda ^a(\alpha )-\lambda ^a_k)c_k^2 \end{aligned} \end{aligned}$$

where in the second equality we used (32).

Furthermore, if $C_1=\lambda ^a(2m-\frac{1}{2})-\lambda ^a(2m-1)$, then

$$\begin{aligned} \sum _{\{k:\ \alpha _k<2m\}}(\lambda ^a(\alpha )-\lambda ^a_k)c_k^2 \ge C_1 \sum _{\{k:\ \alpha _k<2m\}}c_k^2=C_1\Vert {\overline{P}}\Vert _{L^2(\partial B_1,a)} ^2\ge \frac{C_1}{C_0}M^2 \end{aligned}$$

where the last inequality follows by

$$\begin{aligned} \begin{aligned}M^2&\le \left( \sum _{\{k:\ \alpha _k<2m\}}|c_k |\Vert \phi _k \Vert _{L^\infty (\partial B_1)}\right) ^2\le C_0\sum _{\{k:\ \alpha _k<2m\}} |c_k|^2 \\&=C_0\Vert {\overline{P}}\Vert _{L^2(\partial B_1,a)}^2, \end{aligned} \end{aligned}$$

where we used the Cauchy–Schwartz inequality and that all norms in a finite dimensional space are equivalent.

We deduce that

$$\begin{aligned} \begin{aligned}&W_{2m}(\zeta )-(1+\varepsilon )W_{2m}(z)\\ {}&\quad =M^2\Vert h_{2m}\Vert _{L^2(\partial B_1,a)}^2\varepsilon ^2\frac{(\alpha +2m+n+a-1)^2}{n+a+2\alpha -1}-\frac{C_1}{C_0}\frac{ M^2\varepsilon }{n+a+2\alpha -1}\\ {}&\quad \le \frac{M^2\varepsilon }{n+a+2\alpha -1}\left( \Vert h_{2m}\Vert _{L^2(\partial B_1,a)}^2 (4m+n)^2\varepsilon -\frac{C_1}{C_0} \right) <0,\end{aligned} \end{aligned}$$

where we have chosen $\varepsilon =\varepsilon (n,m,a)>0$ small enough. $\square$

## Frequency gap

Once we have proved the epiperimetric inequalities in Theorems 1.1, 1.2, 5.1 and 5.2, the proof of the frequency gap is standard, as done in [8].

First notice that without loss of generality we can consider a $\lambda$-homogeneous solution of (4) (with 0 obstacle). In fact, let $v=u^{x_0}$ be a $\lambda$-homogeneous solution of (5) with obstacle $\varphi \in C^{k,\gamma }(\mathbb {R}^n)$, $k\ge 2$ and $\gamma \in (0,1)$. If $\lambda < k+\gamma$, then $\Phi ^{x_0}(0^+,v)=n+a+2\lambda$ by Remark 2.5. Thus we can consider *z*, the blow-up of *v* at $x_0\in \Gamma (u)$, that is a solution of (4) (with 0 obstacle).

### Proof of Proposition 1.3

*Step 1.* To prove the frequency gap around $1+s$, it is sufficient to check that if $\lambda =1+s+t$ with $t>0$, then $t\ge 1-s$, while if $t<0$, then $t\le -(1-s)$.

Let $c\in H^1(\partial B_1,a)$ be a trace of a $(1+s+t)$-homogeneous solution, say $r^{{1+s}+t}c(\theta )$.

1. If $t>0$, then $W_{1+s}(r^{{1+s}+t}c)=t\Vert c\Vert ^2_{L^2(\partial B_1,a)}>0,$ by (33). Thus, using the epiperimetric inequality for $W_{1+s}$ (Theorem 1.1), we obtain
  $$\begin{aligned} \begin{aligned}0<W_{1+s}(r^{{1+s}+t}c)&\le \left( 1-\frac{1+a}{2n+a+5}\right) W_{1+s}(r^{{1+s}}c)\\&=\left( 1-\frac{1+a}{2n+a+5}\right) \left( 1+\frac{t}{n+2}\right) W_{1+s}(r^{{1+s}+t}c), \end{aligned} \end{aligned}$$
  where in the last equality we used (34). Therefore we deduce that
  $$\begin{aligned} \left( 1-\frac{1+a}{2n+a+5}\right) \left( 1+\frac{t}{n+2}\right) \ge 1, \end{aligned}$$
  which implies that $t\ge 1-s$.
2. If $t<0$, then $W_{{1+s}}(r^{{1+s}+t}c)=t\Vert c\Vert _{L^2(\partial B_1,a)}^2<0$ by (33). Thus, using the epiperimetric inequality for negative energies $W_{1+s}$, i.e. Theorem 5.1, we obtain
  $$\begin{aligned} \begin{aligned}W_{{1+s}}(r^{{1+s}+t}c)&\le \left( 1+\frac{1+a}{2n-a+3}\right) W_{{1+s}}(r^{{1+s}}c)\\&=\left( 1+\frac{1+a}{2n-a+3} \right) \left( 1+\frac{t}{n+2}\right) W_{{1+s}}(r^{{1+s}+t}c), \end{aligned} \end{aligned}$$
  where we used (34). Hence, since we have a negative energy, we get
  $$\begin{aligned} \left( 1+\frac{1+a}{2n-a+3}\right) \left( 1+\frac{t}{n+2}\right) \le 1, \end{aligned}$$
  which gives $$t\le - ({1+s})$$.

*Step 2.* Let $c\in H^1(\partial B_1,a)$ be a trace of a $(2m+t)$-homogeneous solution, say $r^{2m+t}c(\theta )$, with $\Vert c\Vert _{L^2(\partial B_1,a)}^2=1$. It is sufficient to check that if $t>0$, then $t\ge c_m^+$, for some $c_m^+>0$, while if $t<0$, then $t\le - c_m^-$, for some $c_m^->0$.

1. Let $$t>0$$, then
  45 $$\begin{aligned} 0<t=W_{2m}(r^{2m+t}c)\le W_{2m}(r^{2m}c), \end{aligned}$$
  by (33). Using the logarithmic epiperimetric inequality for $W_{2m}$, i.e. Theorem 1.2, and (45), we obtain
  $$\begin{aligned} \begin{aligned}0<W_{2m}(r^{2m+t}c)&\le W_{2m}(\zeta )\le (1-\varepsilon W_{2m}^\beta (r^{2m}c))W_{2m}(r^{2m}c)\\ {}&\le (1-\varepsilon t^\beta )W_{2m}(r^{2m}c)\\ {}&=(1-\varepsilon t^\beta )\left( 1+\frac{t}{n+a+4m-1}\right) W_{2m}(r^{2m+t}c), \end{aligned} \end{aligned}$$
  where in the last equality we used (34). Therefore, since we have a negative energy, we have
  $$\begin{aligned} (1-\varepsilon t^\beta )\left( 1+\frac{t}{n+a+4m-1}\right) \ge 1, \end{aligned}$$
  which gives that $t\ge c_m^+$, for some explicit constant $c_m^+>0$.
2. If $t<0$ then $W_{2m}(r^{2m+t}c)=t<0$ by (33), therefore using the epiperimetric inequality for negative energies $W_{2m}$, i.e. Theorem 5.2, we obtain
  $$\begin{aligned} \begin{aligned}W_{2m}(r^{2m+t}c)&\le (1+\varepsilon )W_{2m}(r^{2m}c)\\ {}&=(1+\varepsilon )\left( 1+\frac{t}{n+a+4m-1}\right) W_{2m}(r^{2m+t}c), \end{aligned} \end{aligned}$$
  where in the last equality we used (34). Thus we get
  $$\begin{aligned} (1+\varepsilon )\left( 1+\frac{t}{n+a+4m-1}\right) \le 1, \end{aligned}$$
  which is $t\le -c_m^-$ for some explicit constant $c_m^->0.$

$$\square $$

## Characterization of blow-ups

The epiperimetric inequality approach allows us to give an alternative proof of the characterization of blow-ups, in the spirit of [8]. For the original proof we refer to [7] and [21].

### Proof of Proposition 1.4

*Step 1.* We want to prove that if *z* is a solution of (4) (with 0 obstacle) and it is $(1+s)$-homogeneous, then $z=Ch_e^s$, $e\in \partial B'_1$ and $C\ge 0$.

In fact, suppose that $c\in H^1(\partial B_1,a)$ is the trace of *z* and let $\zeta$ be the competitor in the epiperimetric inequality for $W_{1+s}$. Then $W_{1+s}(z)=0$, by Proposition 2.12. Therefore

$$\begin{aligned} 0=W_{1+s}(z)\le W_{1+s}(\zeta )\le (1-\kappa )W_{1+s}(z)=0, \end{aligned}$$

i.e. the epiperimetric inequality is an equality. Thus, by Remark 3.2, $c(\theta )=Ch_e^s(\theta )+\phi (\theta )$, for some $e\in \partial B'_1$, $C\ge 0$ and $\phi$ eigenfunction corresponding to eigenvalue $\lambda ^a(2)$ with $\phi \ge 0$ on $B'_1$.

Hence, using Proposition 2.12 with an integration by parts, we get

$$\begin{aligned} \begin{aligned}0&=W_{1+s}(z)=W_{1+s}(Cr^{1+s}h_e^s+r^{1+s}\phi ) =W_{1+s}(r^{1+s}h_e^s)+W_{1+s}(r^{1+s}\phi )\\&\quad +2\left( \int _{B_1}\nabla h_e^s \cdot \nabla (r^{1+s}\phi ) |y|^a\,dX-({1+s}) \int _{\partial B_1} h_e^s\phi |y|^a\,d\mathcal {H}^n\right) \\ {}&\ge W_{1+s}(r^{1+s}h_e^s)+W_{1+s}(r^{1+s}\phi )=W_{1+s}(r^{1+s}\phi )\ge 0,\end{aligned} \end{aligned}$$

where we used (30) and (68). In particular the last inequality is actually an equality. Then, by (30), we get that $\phi \equiv 0$, i.e. $z=Ch_e^s$ for some $C\ge 0$.

*Step 2.* Suppose that *z* is a solution of (4) (with 0 obstacle) and that *z* is 2*m*-homogeneous. Then we claim that $z=p_{2m}$ for some polynomial $p_{2m}$ which is $L_a$-harmonic.

Let $c\in H^1(\partial B_1,a)$ be the trace of *z* and let $\zeta$ be the competitor in the logarithmic epiperimetric inequality for $W_{2m}$. Without loss of generality, we can suppose $\Vert c\Vert _{L^2(\partial B_1,a)}=1$. Hence, by the strong version of the log-epiperimetric inequality 43, we have

$$\begin{aligned} \begin{aligned} 0&=W_{2m}(z)\le W_{2m}(\zeta )\le W_{2m}(z)(1-\varepsilon |W_{2m}(z)|^\beta )-\varepsilon _1\Vert \nabla _\theta \phi \Vert _{L^2(\partial B_1,a)}^{2+2\beta }\\&=-\varepsilon _1\Vert \nabla _\theta \phi \Vert _{L^2(\partial B_1,a)}^{2+2\beta } \end{aligned} \end{aligned}$$

i.e. $$\Vert \nabla _\theta \phi \Vert _{L^2(\partial B_1,a)}=0$$.

Thus $\phi \equiv 0$ and *c* contains only eigenfunctions corresponding to eigenvalues $\lambda ^a_k\le \lambda ^a(2m)$, i.e.

$$\begin{aligned} c(\theta )=\sum _{\{k:\ \alpha _k\le 2m\}}c_k\phi _k. \end{aligned}$$

Using (30), we obtain

$$\begin{aligned} 0=W_{2m}(z)=\frac{1}{n+4m-1}\sum _{\{k:\ \alpha _k\le 2m\}}(\lambda ^a_k-\lambda ^a(2m))c_k^2\le 0, \end{aligned}$$

i.e. the frequencies $\alpha _k<2m$ must vanish. Therefore *c* is an eigenfunction corresponding to eigenvalue $\lambda ^a(2m)$ and it follows that the homogeneous extension *z* is a 2*m*-homogeneous $L_a$-harmonic polynomial. $\square$

## Regularity of $\Gamma _{1+s}(u)$ and structure of $\Gamma _{2m}(u)$

We conclude this paper with the most important application of the epiperimetric inequalities in Theorems 1.1 and 1.2, i.e. the proof of Theorem 1.5. The proof following a standard argument, for instance see [19] for the case $s\in (0,1)$ or [8, 13, 20], for the case $s=\frac{1}{2}.$

In this last section, we recall the results from [19], where the claim (1) was proved in the case $s\in (\frac{1}{2},1)$. The regularity assumption for $\varphi$, that is $\varphi \in C^{k,\gamma }(\mathbb {R}^n)$, allows us to generalize the result to any $s\in (0,1)$ by using the same argument.

Indeed, in [19] it was proved that the function $r\mapsto {\widetilde{W}}^{x_0}_{\lambda }(r,v)+Cr^{2s-1}$ is increasing (with a slight difference in the definition of *v*), where $2s-1>0$. We notice that in the case $\varphi \in C^{k,\gamma }(\mathbb {R}^n)$, we have that the function $r\mapsto {\widetilde{W}}^{x_0}_\lambda (r,v)+Cr^{k+\gamma -\lambda }$ is increasing, with $k+\gamma -\lambda >0$, by (23), with $v=u^{x_0}$ the solution of (5).

For the case (2), we give the complete proof, which is based on similar arguments and uses ideas from [8, 18]. Notice that in the case $\lambda =2m$, the condition $\lambda < k+\gamma$ become $\lambda =2\,m\le k$ since $2\,m$ and *k* are integers.

### Decay of the Weiss’ energy ${\widetilde{W}}$

The first result, that follows by the epiperimetric inequalities, is the decay of the Weiss’ energy ${\widetilde{W}}$ for obstacle $\varphi \not \equiv 0.$

#### Proposition 8.1

(Decay of the Weiss’ energy ${\widetilde{W}}$) Let *u* be a solution of (2) and $v=u^{x_0}$ be the solution of (5) with $x_0\in \Gamma (u)$, $\varphi \in C^{k,\gamma }(\mathbb {R}^n)$, $k\ge 2$ and $\gamma \in (0,1)$. Let $K\subset \Gamma _\lambda (u)\cap \mathbb {R}^{n+1}$ be a compact set.

1. If $\lambda =1+s$, then there is $\alpha \in (0,1)$ such that
  46 $$\begin{aligned} {\widetilde{W}}^{x_0}_{1+s}(r,v)\le Cr^\alpha \end{aligned}$$
  for all $x_0\in \Gamma _{1+s}(u)\cap K$ and for all $r\in (0,r_0)$, for some $r_0>0$.
2. If $\lambda =2\,m\le k$ and $\beta \in (0,1)$ is the constant in Theorem 1.2, then
  47 $$\begin{aligned} {\widetilde{W}}^{x_0}_{2m}(r,v)\le C(- \log (r)^{-\frac{1}{\beta }}) \end{aligned}$$
  for all $x_0\in \Gamma _{2m}(u)\cap K$ and for all $r\in (0,r_0)$, for some $r_0>0$.

#### Proof

Let $c_r$ be the $\lambda$-homogeneous extension of $v_r|_{\partial B_1}$. As in Lemma 5.1 in [19], we obtain

48 $$\begin{aligned} \begin{aligned} \frac{d}{dr}{\widetilde{W}}^{x_0}_\lambda (r,v)\ge \frac{n+a+2\lambda -1}{r} \left( W_\lambda (1,c_r)-{\widetilde{W}}^{x_0}_\lambda (r,v)\right) -Cr^{k+\gamma -\lambda -1}. \end{aligned} \end{aligned}$$

and

49 $$\begin{aligned} W_\lambda (1,c_r)\ge {\widetilde{W}}^{x_0}_\lambda (r,v)-Cr^{k+\gamma -\lambda }. \end{aligned}$$

Now we separate the case $\lambda =1+s$ and $\lambda =2\,m$.

*Step 1.* In the case $\lambda =1+s$, we have that

$$\begin{aligned} \frac{d}{dr}{\widetilde{W}}^{x_0}_{1+s}(r,v)\ge \frac{c}{r}{\widetilde{W}}^{x_0}_{1+s}(r,v)-Cr^{k+\gamma -\lambda -1}, \end{aligned}$$

by (48), (49) and by epiperimetric inequality for $W_{1+s}$ (Theorem 1.1).

Now for all $\alpha \in (0,1)$, we have

$$\begin{aligned}\begin{aligned} \frac{d}{dr}\left( {\widetilde{W}}^{x_0}_{1+s}(r,v)r^{-\alpha }\right)&=\frac{d}{dr}{\widetilde{W}}^{x_0}_{1+s}(r,v)r^{-\alpha }-\alpha {\widetilde{W}}^{x_0}_{1+s}(r,v)r^{-\alpha -1}\\&\ge (c-\alpha ){\widetilde{W}}^{x_0}_{1+s}(r,v)r^{-\alpha -1}-Cr^{k+\gamma -\lambda -1-\alpha }\\&\ge -C(c-\alpha )r^{k+\gamma -\lambda -1-\alpha }-Cr^{k+\gamma -\lambda -1-\alpha }\\&=-Cr^{k+\gamma -\lambda -1-\alpha } \end{aligned} \end{aligned}$$

where we have chosen $\alpha <c$ and we used (25). Integrating from *r* to $r_0$, we obtain (46).

*Step 2.* In the case $\lambda =2m$, since $W_{2\,m}(1,c_r)\ge W_{2\,m}(1,\zeta _r)\ge 0$, we have that

$$\begin{aligned} \begin{aligned} W_{2m}(1,c_r)&\ge W_{2m}(1,\zeta _r)+{\varepsilon }\Theta ^{-\beta } W_{2m}(1,c_r)^{1+\beta }\\ {}&\ge W_{2m}(1,\zeta _r)+{\varepsilon }\Theta ^{-\beta } W_{2m}(1,\zeta _r)^{1+\beta } \end{aligned} \end{aligned}$$

by epiperimetric inequality (Theorem 1.2) for $W_{2m}$ (see Remark 8.2 below). Then

50 $$\begin{aligned} \frac{d}{dr}{\widetilde{W}}^{x_0}_{2m}(r,v)\ge \frac{c}{r} {\widetilde{W}}^{x_0}_{2m}(r,v)^{1+\beta }-Cr^{k+\gamma -\lambda -1}, \end{aligned}$$

where we used (48), (49) and where we suppose ${\widetilde{W}}^{x_0}_{2m}(r,v)\ge 0$, since otherwise we were done.

Let $F(r)=\max \{{\widetilde{W}}^{x_0}_{2\,m}(r,v),\left( \frac{2}{c}\right) ^ {\frac{1}{1+\beta }}Cr^{\frac{k+\gamma -\lambda }{1+\beta }}\}$, then we claim that

$$\begin{aligned} F'(r)\ge \frac{c}{2r}F(r)^{1+\beta }, \end{aligned}$$

for all $r\in (0,r_0)$, up to decreasing $r_0$. In fact, if $F'(r)=\frac{d}{dr}{\widetilde{W}}^{x_0}_{2m}(r,v)$, then

$$\begin{aligned} F'(r)\ge \frac{c}{2r}{\widetilde{W}}^{x_0}_{2m}(r,v)^{1+\beta }=\frac{c}{2r}F(r)^{1+\beta }, \end{aligned}$$

by (50) and since ${\widetilde{W}}^{x_0}_{2\,m}(r,v)\ge \left( \frac{2}{c}\right) ^{\frac{1}{1+\beta }} Cr^{\frac{k+\gamma -\lambda }{1+\beta }}.$ While, if

$$\begin{aligned} F'(r)= \left( \frac{2}{c}\right) ^{\frac{1}{1+\beta }}C\left( \frac{k+\gamma -\lambda }{1+\beta }\right) r^{\frac{k+\gamma -\lambda }{1+\beta }-1}, \end{aligned}$$

then

$$\begin{aligned}\begin{aligned} F'(r)\ge \frac{c}{2}\left( \frac{2}{c}\right) ^{\frac{1}{1+\beta }}Cr^{k+\gamma -\lambda -1}=\frac{c}{2r}F(r)^{1+\beta }, \end{aligned} \end{aligned}$$

since ${\widetilde{W}}^{x_0}_{2\,m}(r,v)\le \left( \frac{2}{c}\right) ^{\frac{1}{1+\beta }} Cr^{\frac{k+\gamma -\lambda }{1+\beta }},$ that is the claim

Now notice that the function $r\mapsto -F(r)^{-\beta }-\frac{c}{2}\beta \log (r)$ is increasing for $r\in (0,r_0)$, by the previous inequality.

Therefore, if we choose $r_0>0$ such that ${\widetilde{W}}_{2m}^{x_0}(r_0,v)\ge 0$, we have that (47) holds, if $r\le r_0^2$.

$$\square $$

#### Remark 8.2

In the Step 2 of the proof of Proposition 8.1, we used the logarithmic epiperimetric inequality for the rescaled $c_r$, but to use Theorem 1.2, we have to check that the conditions (11) hold with $\Theta >0$ that does not depend on *r*.

In fact $\Vert c_r\Vert ^2_{L^2(\partial B_1,a)}=\Vert v_r\Vert ^2_{L^2(\partial B_1,a)}\le \Theta$ as in the proof of Proposition 2.9.

Moreover, by (66)

$$\begin{aligned}\begin{aligned} W_{2m}(c_r)&\le C\Vert \nabla _\theta c_r\Vert ^2 _{L^2(\partial B_1,a)}\le C\Vert \nabla c_r\Vert ^2 _{L^2( B_1,a)}\\ {}&=C(\Vert \nabla _\theta v_r\Vert ^2 _{L^2(\partial B_1,a)}+\lambda ^2\Vert v_r\Vert ^2 _{L^2(\partial B_1,a)})\\ {}&\le C(\Vert \nabla v_r\Vert ^2 _{L^2( B_1,a)}+\lambda ^2\Vert v_r\Vert ^2 _{L^2(\partial B_1,a)})\le \Theta , \end{aligned} \end{aligned}$$

where in the equality we used (65) and in the last inequality we used the boundness of $v_r$ in $H^1(B_1,a)$, as in the proof of Proposition (2.9).

### Decay of homogeneous rescalings

The decay of the Weiss’ energy allows us to prove a decay of the norm in $L^1(\partial B_1,a)$ of the homogeneous rescalings. As a consequence, we get the uniqueness of the homogeneous blow-up.

#### Proposition 8.3

(Decay of homogeneous rescalings) Let *u* be a solution of (2) and $v=u^{x_0}$ be the solution of (5) with $x_0\in \Gamma (u)$, $\varphi \in C^{k,\gamma }(\mathbb {R}^n)$, $k\ge 2$ and $\gamma \in (0,1)$. Let $v^{(\lambda )}_{r,x_0}$ be the homogeneous rescalings from (26), and $K\subset \Gamma _\lambda (u)\cap \mathbb {R}^{n+1}$ be a compact set.

1. If $\lambda =1+s$, then, up to decreasing $\alpha$ in Proposition 8.1, we have
  51 $$\begin{aligned} \int _{\partial B_1} |v^{(1+s)}_{r,x_0}-v^{(1+s)}_{r',x_0}||y|^a\,d\mathcal {H}^n\le Cr^\alpha \end{aligned}$$
  for all $x_0\in \Gamma _{1+s}(u)\cap K$ and for all $0<r'<r<r_0$, for some $r_0>0$.
2. If $\lambda =2m\le k$, and $\beta \in (0,1)$ is the constant in Theorem 1.2, then
  52 $$\begin{aligned} \int _{\partial B_1} |v^{(2m)}_{r,x_0}-v^{(2m)}_{r',x_0}||y|^a\,d\mathcal {H}^n\le C(- \log (r)^{-\frac{1}{2\beta }} )\end{aligned}$$
  for all $x_0\in \Gamma _{2m}(u)\cap K$ and for all $0<r'<r<r_0$, for some $r_0>0$.

In particular, the homogeneous blow-up $v^{(\lambda )}_{0,x_0}$ is unique and the whole sequence $v^{(\lambda )}_{r,x_0}$ converges to $v^{(\lambda )}_{0,x_0}$ as $r\rightarrow 0^+$.

#### Proof

Dropping the dependence on *x* and $\lambda$, for $0<r'<r<r_0$, where $r_0$ is as in the previous Proposition, using (23), we get

$$\begin{aligned} \int _{\partial B_1} |&v_{r}-v_{r'}||y|^a\,d\mathcal {H}^n\le C\log \left( \frac{r}{r'}\right) ^\frac{1}{2}\left( {\widetilde{W}}^{x_0}_\lambda (r,v)+r^{k+\gamma -\lambda }\right) ^\frac{1}{2}, \end{aligned}$$

as in [19].

The conclusion follows by a dyadic decomposition as in [13, 20] or [8], and by using (46) for $\lambda =1+s$, and (47) for $\lambda =2m$. $\square$

### Non-degeneracy of homogeneous blow-up

Another consequence of the decay of the Weiss’ energy is the non-degeneracy of the homogeneous blow-ups, i.e. the homogeneous blow-ups cannot vanish identically.

#### Proposition 8.4

(Non-degeneracy of homogeneous blow-up) Let *u* be a solution of (2) and $v=u^{x_0}$ be the solution of (5) with $x_0\in \Gamma (u)$, $\varphi \in C^{k,\gamma }(\mathbb {R}^n)$, $k\ge 2$ and $\gamma \in (0,1)$. If $\lambda =1+s$ or $\lambda =2m\le k$, then

$$\begin{aligned} \begin{aligned} H^{x_0}(r)\ge H_0^{x_0}r^{n+a+2\lambda } \end{aligned} \end{aligned}$$

for all $x_0\in \Gamma _\lambda (u)$ and for all $r\in (0,r_0)$, for some $r_0>0$, where

$$\begin{aligned} H_0^{x_0}:=\lim _{r\rightarrow 0^+}\frac{H^{x_0}(r)}{r^{n+a+2\lambda }}>0. \end{aligned}$$

In particular the homogeneous blow-up $v^{(\lambda )}_{0,x}$ is non-trivial, since

$$\begin{aligned} \Vert v^{(\lambda )}_{0,x_0} \Vert _{L^2(\partial B_1,a)}=H_0^{x_0}>0. \end{aligned}$$

#### Proof

Note that the inequality follows by the proof of (14), in fact we can obtain that the function $r\mapsto \frac{H^{x_0}(r)}{r^{n+a+2\lambda }}$ is increasing for *r* small enough. Hence it is sufficient to prove that $H_0^{x_0}>0$.

If by contradiction $H_0^{x_0}=0$, we can arguing as in Lemma 7.2 in [8]. $\square$

### Regularity of blow-ups

Roughly speaking, we prove the regularity of the map that to any point $x\in \Gamma _\lambda (u)\cap K$ associates the homogeneous blow-up of $v=u^{x}$ at *x*, where $\lambda =1+s$ or $\lambda =2m$. We are able to prove the regularity with an explicit modulus of continuity that depend on the right-hand side of (51) and (52).

#### Proposition 8.5

Let *u* be a solution of (2) and $v=u^{x}$ be the solution of (5) with $x\in \Gamma _\lambda (u)$, $\varphi \in C^{k,\gamma }(\mathbb {R}^n)$, $k\ge 2$ and $\gamma \in (0,1)$. Let $v_{0,x}^{(\lambda )}$ be the $\lambda$-homogeneous blow-up of $v=u^{x}$ at *x* and $K\subset \Gamma _\lambda (u)\cap \mathbb {R}^{n+1}$ be a compact set.

1. If $$\lambda =1+s$$ and
  $$\begin{aligned} v_{0,x}^{(1+s)}=\lambda _x h_{e(x)}^s, \end{aligned}$$
  with $\lambda _x>0$, $e(x)\in \partial B'_1$ and $h_e^s$ as in (12), then up to decreasing $\alpha$ in Proposition 8.3, it holds
  53 $$\begin{aligned} |\lambda _{x_1} -\lambda _{x_2}|\le C|x_1-x_2|^\alpha \end{aligned}$$
  and
  54 $$\begin{aligned} |e(x_1) -e(x_2)|\le C|x_1-x_2|^\alpha \end{aligned}$$
  for all $$x_1,x_2\in \Gamma _{1+s}(u)\cap K$$.
2. If $$\lambda =2m\le k$$ and
  $$\begin{aligned} v_{0,x}^{(2m)}=\lambda _xp_x \end{aligned}$$
  with $\lambda _x>0$ and $p_x$ a 2*m*-homogeneous polynomial such that $\Vert p_x\Vert _{L^2(\partial B_1,a)}=1$, then
  55 $$\begin{aligned} |\lambda _{x_1} -\lambda _{x_2}|\le C(-\log (|x_1-x_2|)^{-\frac{1-\beta }{2\beta }})\end{aligned}$$
  and
  56 $$\begin{aligned} \Vert p_{x_1} -p_{x_2}\Vert _{L^\infty ( B_1)}\le C(-\log (|x_1-x_2|)^{-\frac{1-\beta }{2\beta }})\end{aligned}$$
  for all $$x_1,x_2\in \Gamma _{2m}(u)\cap K$$.

#### Proof

We sketched the proof, similar to the one done in [19].

First note that the characterization of blow-up and $\lambda _x>0$ follows by Proposition 1.4 and Proposition 8.4.

Secondly, for some dimensional constant $c_0>0$, we have

57 $$\begin{aligned} c_0\frac{H^{x_i}(r)}{r^{n+a+2\lambda }}-\lambda _{x_i}^2\le C\omega _\alpha (r), \end{aligned}$$

where we denote by

$$\begin{aligned} \omega _\alpha (r)={\left\{ \begin{array}{ll} r^\alpha &{} \text{ if } \lambda =1+s\\ (-\log (r)^{-\frac{1-\beta }{2\beta }}) &{} \text{ if } \lambda =2m \end{array}\right. } \end{aligned}$$

the modulus of continuity. We indicate explicitly the dependence on $\alpha$ since it must be reduced to obtain the final claim.

Let now $x_1,x_2\in \Gamma _\lambda (u)\cap K$ and $r=|x_1-x_2|^\sigma$ with $\sigma \in (0,1)$ to choose. Let $w_r$ be the homogeneous rescalings in (26) of $w=u-\varphi$. Using the regularity of the solution and $\nabla w(x_2,0)=0$, we deduce that

58 $$\begin{aligned} \begin{aligned}&\int _{\partial B_1} |w_{r,x_1}-w_{r,x_2}||y|^a\,d\mathcal {H}^n\le C\omega _{1-\sigma }(|x_1-x_2|), \end{aligned} \end{aligned}$$

where we can choose, for example, $\sigma =\frac{1}{2m}$ for $\lambda =2m$.

Let $Q ^{x_i}$ as in Lemma (9.7), we have

$$\begin{aligned} \begin{aligned} \int _{\partial B_1} |Q^{x_1}_{r,x_1}&- Q^{x_2}_{r,x_2}||y|^a\,d\mathcal {H}^n\\&\le \int _{\partial B_1} |Q^{x_1}_{r,x_1}- Q^{x_2}_{r,x_1}||y|^a\,d\mathcal {H}^n+\int _{\partial B_1} |Q^{x_2}_{r,x_1}- Q^{x_2}_{r,x_2}||y|^a\,d\mathcal {H}^n\\ {}&\le C\omega _{k+\gamma -\lambda }(r)+ C\omega _{1-\sigma }(|x_1-x_2|), \end{aligned} \end{aligned}$$

where in the last inequality we have used Lemma 9.7 for the first term, and the same computation in (58) for the second term. Therefore, recalling the definition of $u^{x_i}$ that is a solution of (4), we deduce that

59 $$\begin{aligned} \int _{\partial B_1} |u^{x_1}_{r,x_1}-u^{x_2}_{r,x_2}||y|^a\,d\mathcal {H}^n\le C\omega _{1-\sigma }(|x_1-x_2|), \end{aligned}$$

if $\sigma \in (0,1)$ such that $(k+\gamma -\lambda )\sigma =1-\sigma$ in the case $\lambda =1+s$.

By the estimates (57) and (59) (with the regularity of the solution), we obtain

$$\begin{aligned} |\lambda _{x_1}&-\lambda _{x_2}|\le C\omega _{\delta }(|x_1-x_2|), \end{aligned}$$

if we choose $\delta =\alpha \sigma \wedge (1-\sigma )$.

Furthermore, we denote by $u_{0,x_i}$ the homogeneous blow-up of the rescalings in (26) of the function $u^{x_i}$. By (51), (52) and (59), we get

60 $$\begin{aligned} \begin{aligned} \int _{\partial B_1} |u_{0,x_1}-u_{0,x_2}|&|y|^a\,d\mathcal {H}^n\le \omega _{\delta }(|x_1-x_2|), \end{aligned} \end{aligned}$$

since $$\delta =\alpha \sigma \wedge (1-\sigma )$$.

Hence, by the homogeneity of $u_{0,x_1}-u_{0,x_2}$ and by Proposition 9.6, it follows that

61 $$\begin{aligned} \begin{aligned}\int _{\partial B'_1} |u_{0,x_1}-u_{0,x_2}|^2\,d\mathcal {H}^{n-1}\le \omega _{\delta }(|x_1-x_2|) \end{aligned} \end{aligned}$$

where in the last inequality we used (60) (with the regularity of the solution).

Using (53), (55) and (61), we get

62 $$\begin{aligned} \begin{aligned} \int _{\partial B'_1} \left|\frac{u_{0,x_1}}{\lambda _{x_1}}-\frac{u_{0,x_2}}{\lambda _{x_2}}\right|\,d\mathcal {H}^{n-1}\le C\omega _{\delta }^\frac{1}{2}(|x_1-x_2|), \end{aligned} \end{aligned}$$

where we used the $\omega$-continuity of the function $x\mapsto \lambda _x$ for $x\in K$ to estimate from below $\lambda _{x_1}$.

Now, by (62) and the definition of $h_e^s$ in (12), for $\lambda =1+s$ we have

$$\begin{aligned} \int _{\partial B'_1}|(x\cdot e_{x_1})_+^{1+s}-(x\cdot e_{x_2})_+^{1+s}|\,d\mathcal {H}^{n-1}\le Cr^{\frac{\delta }{2}}, \end{aligned}$$

which implies (54).

Finally, for $\lambda =2m$, since all norms in a finite dimensional space are equivalent, we get

$$\begin{aligned} \Vert p_{x_1} -p_{x_2}\Vert _{L^\infty ( B_1)}\le C \int _{\partial B_1}|p_{x_1}-p_{x_2}||y|^a\,d\mathcal {H}^{n}. \end{aligned}$$

By a similar computation as in (62), we obtain

$$\begin{aligned} \int _{\partial B_1}|p_{x_1}-p_{x_2}||y|^a\,d\mathcal {H}^{n}\le C (-\log (|x_1-x_2|)^{-\frac{1-\beta }{2\beta }}), \end{aligned}$$

where we used (60), then we conclude (56) $\square$

### Proof of Theorem 1.5

For the regularity of $\Gamma _{1+s}(u)$ and the structure of $\Gamma _{2m}(u)$ for $2m\le k$ we can proceed with a standard argument, as in [8, 13, 18–20].

## Data Availability

This manuscript has no associated data.

## Appendices

In the following proposition, we see that a solution *u* of (4) (with 0 obstacle) is a solutions of $L_a u=f$ in the whole ball $B_1$ with right-hand side *f* which is a measure depending on *u*.

### Proposition 9.1

Let $u\in H^1(B_1,a)$ be a solution of $L_au=0$ in $B_1^+$ and even in the *y* direction. Then

63 $$\begin{aligned} \text{ div }(|y|^a\nabla u)=2\lim _{y\rightarrow 0^+}(|y|^a\partial _y u)\mathcal {H}^n|_{\{y=0\}}.\end{aligned}$$

### Proof

This is a simple consequence of an integration by parts, but for the sake of completeness we give the complete proof.

We compute $\text{ div }(|y|^a\nabla u)$ in distributional sense, as following

$$\begin{aligned} \begin{aligned}<-\text{ div }(|y|^a\nabla u),\eta >&=2\int _{B_1^+} \nabla u \cdot \nabla \eta |y|^a\,dX\\ {}&=2 \lim _{\varepsilon \rightarrow 0^+}\int _{B_1^+\cap \{y\ge \varepsilon \}} \nabla u \cdot \nabla \eta |y|^a\,dX\\&=2\lim _{\varepsilon \rightarrow 0^+}\int _{B^+_1\cap \{y=\varepsilon \}}\eta \partial _\nu u{\varepsilon }^a\,d\mathcal {H}^n\\&=-2\int _{B'_1}\eta \lim _{{\varepsilon }\rightarrow 0^+}(\partial _y u(x',{\varepsilon }){\varepsilon }^a)\,d\mathcal {H}^n, \end{aligned} \end{aligned}$$

where we used the integration by parts

$$\begin{aligned} \begin{aligned} \int _{B_1}\nabla u\cdot \nabla \eta |y|^a\,dX&=\int _{\partial B_1}\eta \partial _\nu u|y|^a\mathcal {H}^n-\int _{B_1}\text{ div }(|y|^a\nabla u)\eta \,dX \end{aligned} \end{aligned}$$

and $$-\text{ div }(|y|^a\nabla u)=0$$ in $$B_1^+\cap \{y\ge \varepsilon \}$$. $$\square $$

In the following lemma we prove some useful identities.

### Lemma 9.2

Let $w\in H^1(B_1,a)$ be $\lambda$-homogeneous. Then

64 $$\begin{aligned}{} & {} \int _{B_1} w|y|^a\,dX=\frac{1}{n+a+\lambda +1}\int _{\partial B_1} w|y|^a\,d\mathcal {H}^n, \end{aligned}$$

65 $$\begin{aligned}{} & {} \Vert \nabla w\Vert ^2 _{L^2( B_1,a)}=\frac{1}{n+a+2\lambda -1}\Vert \nabla _\theta w\Vert ^2 _{L^2(\partial B_1,a)}+\frac{\lambda ^2}{n+a+2\lambda -1}\Vert w\Vert ^2 _{L^2(\partial B_1,a)}\qquad \quad \end{aligned}$$

and

66 $$\begin{aligned} W_\lambda (w)=\frac{1}{n+a+2\lambda -1}\left( \Vert \nabla _\theta w\Vert ^2 _{L^2(\partial B_1,a)}-\lambda (\lambda +n+a-1)\Vert w\Vert ^2 _{L^2(\partial B_1,a)}\right) , \end{aligned}$$

where $\nabla _\theta w(x)$ is the gradient of *w* on $\partial B_1$.

Moreover, if *w* is also a solution of (4) (with 0 obstacle), then

67 $$\begin{aligned} \Vert \nabla _\theta w\Vert ^2 _{L^2(\partial B_1,a)}=\lambda (\lambda +n+a-1)\Vert w\Vert ^2 _{L^2(\partial B_1,a)}, \end{aligned}$$

in particular

68 $$\begin{aligned} W_\lambda (w)=0. \end{aligned}$$

### Proof

The proof is a straightforward computation. $\square$

The following lemma allows us to reduce the problem (2) for obstacle $\varphi \not \equiv 0$, to the problem (5) with 0 obstacle and right-hand side *h*.

### Lemma 9.3

Let $q_k(x)$ be an homogeneous polynomial of degree *k*. Then there is a unique ${\widetilde{q}}_k(x,y)$ homogeneous polynomial of degree *k* such that

69 $$\begin{aligned} {\left\{ \begin{array}{ll} L_a {\widetilde{q}}_k(x,y)=0&{} \text{ in } \mathbb {R}^{n+1}\\ {\widetilde{q}}_k(x,y)={\widetilde{q}}_k(x,-y)&{} \text{ in } \mathbb {R}^{n+1}\\ {\widetilde{q}}_k(x,0)=q_k(x) &{} \text{ in } \mathbb {R}^n\\ \end{array}\right. } \end{aligned}$$

In particular, an homogeneous polynomial of degree *k*, that satisfies (69) and vanishes identically on $\{y=0\}$, must vanish identically on $\mathbb {R}^{n+1}$.

### Proof

See Lemma 5.2 in [21]. $\square$

The following theorem is a generalization of Poincaré Theorem in weighted Sobolev space $H^1(B_R,a).$

### Theorem 9.4

Let $$w\in H^1(B_R,a)$$. Then

70 $$\begin{aligned} \int _{B_R}w^2|y|^a\,dX\le C\left( \int _{B_R}|\nabla w|^2|y|^a\,dX+\int _{\partial B_R}w^2|y|^a\,d\mathcal {H}^n\right) ,\end{aligned}$$

with $$C=C(n,a)>0.$$

### Proof

See Lemma 2.10 in [7]. $\square$

We recall the following generalization of the Liouville’s Theorem for entire $L_a$-harmonic functions.

### Theorem 9.5

(Liouville Theorem) Let *w* be a global solution of $L_aw(x,y)=0$ for $(x,y)\in \mathbb {R}^n\times \mathbb {R}$ such that *w* is even in the *y* direction and

$$\begin{aligned} |w(x,y)|\le C |(x,y)|^\alpha . \end{aligned}$$

Then, *w* is a polynomial.

### Proof

See Lemma 2.7. in [7]. $\square$

In the following Proposition, we show an embedding from $H^1(B_1,a)$, the weighted Sobolev space in $B_1$, to $L^2(B'_1)$, the Lebesgue space on $B'_1$.

### Proposition 9.6

If $a\in (-1,1)$, then there is a bounded operator $T:H^1(B_1,a)\rightarrow L^2(B'_1)$, i.e.

$$\begin{aligned} \Vert w \Vert _{L^2(B'_1)}\le C\Vert w \Vert _{H^1(B_1,a)}, \end{aligned}$$

for all $w\in H^1(B_1,a)$. Moreover

$$\begin{aligned}\begin{aligned}\Vert \phi \Vert _{L^2(\partial B'_1)}\le C\Vert \phi \Vert _{H^1(\partial B_1,a)} \end{aligned} \end{aligned}$$

for all $$\phi \in H^1(\partial B_1,a)$$.

### Proof

See Theorem 2.8. in [22]. $\square$

### Lemma 9.7

Let $\varphi \in C^{k,\gamma }(\mathbb {R}^n)$ and $x_1,x_2\in \mathbb {R}^n$. Let $Q ^{x_i}(x,y)={\widetilde{q}}_k^{x_i}(x,y)-q_k^{x_i}(x)$, where $q_k^{x_i}$ is the *k*-th Taylor polynomial of $\varphi$ at $x_i$ and ${\widetilde{q}}_k^{x_i}$ is the extension according to Lemma 9.3. If $r=|x_1-x_2|^\sigma$ for some $\sigma \in (0,1)$, then

71 $$\begin{aligned} \int _{\partial B_1} |Q^{x_1}_{r,x_1}-Q^{x_2}_{r,x_1}||y|^a\,d\mathcal {H}^n\le Cr^{k+\gamma -\lambda }, \end{aligned}$$

where $Q^{x_i}_{r,x_1}$ are the rescalings of $Q^{x_i}$ as in (26).

### Proof

We denote by *q* the function $q_k$ and we denote by ${\widetilde{q}}$ the function ${\widetilde{q}}_k$. Moreover we consider $q^{x_i}_{r,x_i}$ the rescalings of $q^{x_i}$ as in (26).

Notice that, by $C^{k,\gamma }$-regularity of $\varphi ,$ we get

$$\begin{aligned} \Vert \varphi -q^{x_1}\Vert _{L^\infty (B_r(x_1))}\le C{r}^{k+\gamma } \end{aligned}$$

and

$$\begin{aligned} \Vert \varphi -q^{x_2}\Vert _{L^\infty (B_r(x_1))}\le \Vert \varphi -q^{x_2}\Vert _{L^\infty (B_{2r}(x_2))}\le C{r}^{k+\gamma }, \end{aligned}$$

then

72 $$\begin{aligned} \Vert q^{x_1}-q^{x_2}\Vert _{L^\infty (B_r(x_1))}\le Cr^{k+\gamma }. \end{aligned}$$

We deduce that

$$\begin{aligned} \begin{aligned} \int _{\partial B_1} |q^{x_1}_{r,x_1}-q^{x_2}_{r,x_1}|&|y|^a\,d\mathcal {H}^n\\ {}&=\frac{1}{r^\lambda }\int _{\partial B_1} |q^{x_1}(x_1+rx,ry)-q^{x_2}(x_1+rx,ry)||y|^a\,d\mathcal {H}^n\\ {}&=\frac{1}{r^{\lambda +n+a}}\int _{\partial B_r} |q^{x_1}(z)-q^{x_2}(z)||z_{n+1}|^a\,d\mathcal {H}^n\\&\le \frac{C}{r^\lambda }\Vert q^{x_1}-q^{x_2}\Vert _{L^\infty (\partial B_r(x_1))}\le Cr^{k+\gamma -\lambda }, \end{aligned} \end{aligned}$$

where we used (72) in the last inequality.

Notice that the space of $L_a$-harmonic polynomials in $\mathbb {R}^{n+1}$ of degree *k* which are even in the *y* direction is a finite dimensional space. Then

$$\begin{aligned} \Vert {\widetilde{p}}\Vert _{L^1(B_1,a)}\le C\Vert p\Vert _{L^1(B'_1,a)} \end{aligned}$$

for each *p* which is a polynomial of degree *k*, $L_a$-harmonic and even in the *y* direction. Notice that the right-hand side is a norm by Lemma 9.3.

Consider the operation of extension as in Lemma 9.3 and the operation of rescaling as in (26). These operations commute, by the explicit formulation of the extension, defined in Lemma 5.2 in [21].

Therefore, we have

$$\begin{aligned} \begin{aligned} \int _{\partial B_1} |{\widetilde{q}}^{x_1}_{r,x_1}-\widetilde{q}^{x_2}_{r,x_1}||y|^a\,d\mathcal {H}^n&\le \int _{B_1} |{\widetilde{q}}^{x_1}_{r,x_1}-{\widetilde{q}}^{x_2}_{r,x_1}||y|^a\,dX\\ {}&\le C\int _{B'_1} |q^{x_1}_{r,x_1}- q^{x_2}_{r,x_1}||y|^a\,d\mathcal {H}^{n}\\ {}&\le C\int _{B_1} |q^{x_1}_{r,x_1}- q^{x_2}_{r,x_1}||y|^a\,dX\\ {}&\le \frac{C}{r^\lambda }\Vert q^{x_1}-q^{x_2}\Vert _{L^\infty (B_r(x_1))}\le C r^{k+\gamma -\lambda }, \end{aligned} \end{aligned}$$

by (72), which concludes the proof. $\square$

## Footnotes

1 Only relevant if $W(z)<0$.

2 Only relevant if $W(z)<0$.

## References

[1] Athanasopoulos I, Caffarelli L (2004). Optimal regularity of lower dimensional obstacle problems. Zap. Nauchn. Sem. S.-Peterburg Otdel. Mat. Inst. Steklov..

[2] Athanasopoulos I, Caffarelli L, Salsa S (2008). The structure of the free boundary for lower dimensional obstacle problems. Am. J. Math.

[3] Banerjee A, Buseghin F, Garofalo N (2022). The thin obstacle problem for some variable coefficient degenerate elliptic operators. Nonlinear Anal..

[4] Barrios B, Figalli A, Ros-Oton X (2018). Global regularity for the free boundary in the obstacle problem for the fractional Laplacian. Am. J. Math..

[5] Carducci M (2023). Optimal regularity of the thin obstacle problem by an epiperimetric inequality. Ann. Mat. Pura e Appl. (1923 -).

[6] Caffarelli L, Silvestre L (2007). An extension problem related to the fractional Laplacian. Commun. Partial Differ. Equ..

[7] Caffarelli L, Salsa S, Silvestre L (2008). Regularity estimates for the solution and the free boundary to the obstacle problem for the fractional Laplacian. Invent. Math..

[8] Colombo M, Spolaor L, Velichkov B (2020). Direct epiperimetric inequalities for the thin obstacle problem and applications. Commun. Pure Appl. Math..

[9] Danielli D, Salsa S (2018). Obstacle problems involving the fractional Laplacian. Recent Dev. Nonlocal Theory.

[10] Fefferman C (2009). Extension of $${C}^{m,\omega }-$$smooth functions by linear operators. Rev. Mat. Iberoam..

[11] Fernandez-Real X  (2022). The thin obstacle problem: a survey. Publ. Mat..

[12] Fernández-Real X, Ros-Oton X (2021). Free boundary regularity for almost every solution to the Signorini problem. Arch. Ration. Mech. Anal..

[13] Focardi M, Spadaro E (2016). An epiperimetric inequality for the thin obstacle problem. Adv. Differ. Equ..

[14] Focardi M, Spadaro E (2018). On the measure and the structure of the free boundary of the lower dimensional obstacle problem. Arch. Ration. Mech. Anal..

[15] Focardi M, Spadaro E (2020). The local structure of the free boundary in the fractional obstacle problem. Adv. Calc. Var..

[16] Fernández-Real X, Torres-Latorre C (2023). Generic regularity of free boundaries for the thin obstacle problem. Adv. Math..

[17] Geraci F (2019). An epiperimetric inequality for the lower dimensional obstacle problem. ESAIM: COCV.

[18] Garofalo N, Petrosyan A (2009). Some new monotonicity formulas and the singular set in the lower dimensional obstacle problem. Invent. Math..

[19] Garofalo N, Petrosyan A, Pop C, Smit Vega Garcia M (2017). Regularity of the free boundary for the obstacle problem for the fractional Laplacian with drift. Ann. Inst. Henri Poincaré Anal. Non Linéaire.

[20] Garofalo N, Petrosyan A, Smit Vega Garcia M (2016). An epiperimetric inequality approach to the regularity of the free boundary in the Signorini problem with variable coefficients. J. Math. Pures Appl..

[21] Garofalo N, Ros-Oton X (2020). Structure and regularity of the singular set in the obstacle problem for the fractional Laplacian. Rev. Mat. Iberoam..

[22] Nekvinda A (1993). Characterization of traces of the weighted Sobolev space $${W}^{1, p}({\Omega }, d_m^\varepsilon )$$ on $${M}$$. Czechoslov. Math. J..

[23] Petrosyan, A., Shahgholian, H., Uraltseva, N.: Regularity of free boundaries in obstacle-type problems, vol. 136 of Graduate Studies in Mathematics. American Mathematical Society (2012)

[24] Ros-Oton, X.: Integro-differential elliptic equations. To appear in Progress in Mathematics, Birkhauser (2024)

[25] Silvestre L  (2007). Regularity of the obstacle problem for a fractional power of the Laplace operator. Commun. Pure Appl. Math.

[26] Whitney A (1934). Analytic Extensions of Differentiable Functions Differentiable in Closed Sets.

